# Pentapartite fractionation of particles in oral fluids by differential centrifugation

**DOI:** 10.1038/s41598-021-82451-6

**Published:** 2021-02-08

**Authors:** Chiho Hiraga, Satoshi Yamamoto, Sadamitsu Hashimoto, Masataka Kasahara, Tamiko Minamisawa, Sachiko Matsumura, Akira Katakura, Yasutomo Yajima, Takeshi Nomura, Kiyotaka Shiba

**Affiliations:** 1 Division of Protein Engineering, Cancer Institute, Japanese Foundation for Cancer Research, Ariake 3-8-31, Koto-ku, Tokyo, 135-8550 Japan; 2 Department of Oral Oncology, Oral and Maxillofacial Surgery, Tokyo Dental College, 5-11-13 Sugano, Ichikawa, Chiba 272-8513 Japan; 3 Department of Pharmacology, Tokyo Dental College, 2-1-14 Misaki-cho, Chiyoda-ku, Tokyo, 101-0061 Japan; 4 Laboratory of Biology, Tokyo Dental College, 2-9-7 Kanda-Surugadai, Chiyoda-ku, Tokyo, 101-0062 Japan; 5 Department of Oral Pathobiological Science and Surgery, Tokyo Dental College, 2-9-18 Misaki-cho, Chiyoda-ku, Tokyo, 101-0061 Japan; 6 Department of Oral Implantology, Tokyo Dental College, 2-9-18 Misaki-cho, Chiyoda-ku, Tokyo, 101-0061 Japan

**Keywords:** Oral cancer detection, Membrane proteins, Mitochondrial proteins, DNA, Autophagy, Multivesicular bodies

## Abstract

Oral fluids (OFs) contain small extracellular vesicles (sEVs or exosomes) that carry disease-associated diagnostic molecules. However, cells generate extracellular vesicles (EVs) other than sEVs, so the EV population is quite heterogeneous. Furthermore, molecules not packaged in EVs can also serve as diagnostic markers. For these reasons, developing a complete picture of particulate matter in the oral cavity is important before focusing on specific subtypes of EVs. Here, we used differential centrifugation to fractionate human OFs from healthy volunteers and patients with oral squamous cell carcinoma into 5 fractions, and we characterized the particles, nucleic acids, and proteins in each fraction. Canonical exosome markers, including CD63, CD9, CD133, and HSP70, were found in all fractions, whereas CD81 and AQP5 were enriched in the 160K fraction, with non-negligible amounts in the 2K fraction. The 2K fraction also contained its characteristic markers that included short derivatives of EGFR and E-cadherin, as well as an autophagosome marker, LC3, and large multi-layered vesicles were observed by electronic microscopy. Most of the DNA and RNA was recovered from the 0.3K and 2K fractions, with some in the 160K fraction. These results can provide guideline information for development of purpose-designed OF-based diagnostic systems.

## Introduction

The development of oral fluid (OF)-based diagnostic systems has been attracting attention because of the noninvasive nature of this method of specimen collection^1–4^. The potential use of OFs in diagnoses has been proposed for oral-associated lesions, such as Sjögren’s syndrome and oral cancer, as well as for systemic diseases and cancers distant to the oral cavity^1–4^. Among the oral diseases, our focus has been on oral squamous cell carcinoma (OSCC), the predominant form of oral cancer, for the following reasons: (i) the number of patients with OSCC has been increasing^5^ and (ii) the current staging of OSCC is incomplete and it frequently reoccurs in patients diagnosed at an early stage of OSCC^6^. We hypothesize that characterization of extracellular vesicles (EVs) from OFs could provide valuable clinical information because these vesicles hold molecules responsible for cancer progression^7^. Therefore, EV analysis with OF-based diagnostic systems could enable early detection of OSCC and/or provide auxiliary information for patient stratification.

The name EV is a collective term for a group of membranous vesicles that are secreted from cells^8^. Recent studies have revealed that cells produce various types of EVs by distinct generation mechanisms^9,10^. One type of EV is generated from endosomal multivesicular bodies (MVBs) and is often called an exosome^11^. Exosomes have a relatively small size (40–100 nm), but small EVs (sEVs) are produced by routes other than the MVB pathway, as they can be generated by direct budding of plasma membrane, detachment from cellular protrusions, and regulated cell death^9,10^. These other pathways can also release large EVs ranging in size from 100 nm to several μm.

Previous work on EVs in this century have mostly focused on the smaller EV; however, recent studies have revealed that the larger EV also have important biological activities^12–18^. For this reason, knowledge of the complete range of EVs contained in OFs becomes important for developing OF-based diagnostic systems. In the present study, we have used differential centrifugation to fractionate the particles contained in OFs, and we have characterized the contents of the different fractions. We then compared the differences in the distributions of cancer-associated molecules between healthy donors and patients with OSCC to narrow down the subclasses of EVs that carry diagnostic information for OSCC.

## Results

### Pentapartite fractionation of oral fluids by differential centrifugation

**Figure 1:**
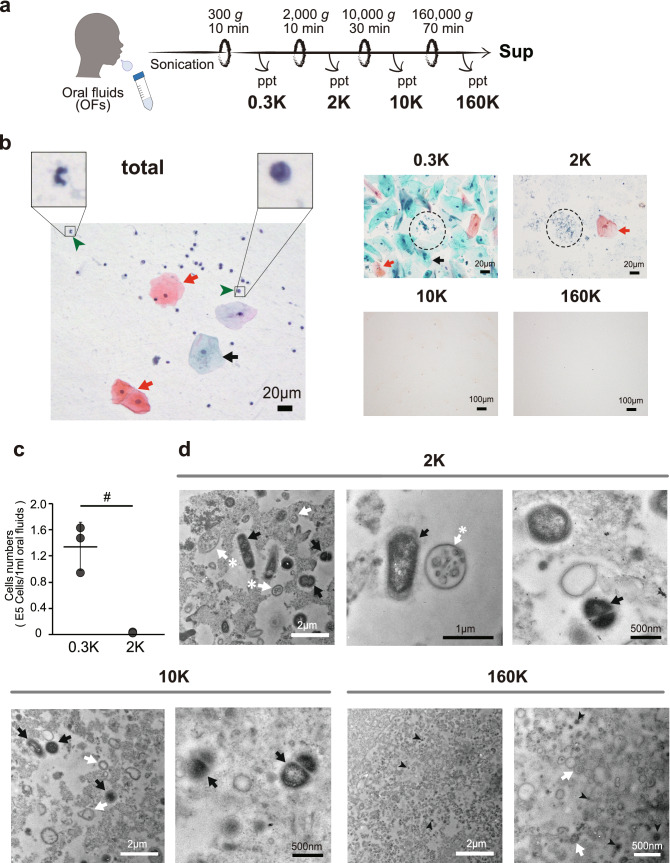
Characterizations of the particulate matter contained in oral fluids (OFs). (**a**) Scheme of the pentapartite fractionation of OFs by differential centrifugation. Total OFs were sequentially centrifuged with increasing *g* force to prepare the 0.3K, 2K, 10K, and 160K pellets and the last supernatant (Sup) fraction. (**b**) Microscope images of Papanicolaou (Pap)-stained samples from the unfractionated OFs (total) and the 0.3K, 2K, 10K, and 160K fractions. Red and black arrows show superficial and intermediate epithelial squamous cells, respectively. Green arrowheads represent examples of leucocyte (Leu). Bacterial-like particles are circled by dash lines. Bars represent 20 µm and 100 µm for the total, 0.3K and 2K images and the 10K and 160K images, respectively. (**c**) Quantification of the cells contained in the 0.3K and 2K fractions by trypan blue staining. OFs were obtained from 3 healthy donors. Dots indicate the mean value of the three measurements for each subject. The mean values for the 3 healthy donors are represented as solid horizontal lines. The two-tailed unpaired *t*-test was used to evaluate statistical significance. # represents p < 0.05. (**d**) Transmission electron microscopy (TEM) images after positive staining of thin sections of the 2K, 10K, and 160K pellets (see “Methods”). Large particles with multiple bilayers (white arrows) are abundant in the 0.3K and 2K fractions; some of these contained intraluminal vesicle structures (labeled with asterisks). The 2K and 10K fractions also contained high-density (high contrast in TEM) particles, which likely represent oral bacteria cells (black arrows). The 160K fraction consisted of tiny particles, many of which has a vesicle structure with a single bilayer. As reported in other body fluids^24–27^, some of these sEV had multiple bilayers (white arrows). The 160K fraction also contained tiny particles with high densities (black arrowheads), which may represent EVs from bacteria or tiny bacteria. Additional images are shown in Figure S2.

An overall picture of the particulate matter present in the oral cavity was obtained by sequential centrifugation (300*g* × 10 min, 2000*g* × 10 min, 10,000*g* × 30 min and 160,000*g* × 70 min) of OFs from five healthy volunteers to obtain 0.3K, 2K, 10K, 160K, and Sup pentapartite fractions (Fig. 1a). We first investigated the cellular components by Papanicolaou (Pap) staining, a widely used protocol in cytologic diagnosis^19^. As shown in Fig. 1b, unfractionated OFs contain many large desquamative squamous cells. (red and black arrows) and small immune cells (green arrowheads), and these were mostly recovered in the 0.3K fraction, with a much smaller portion retained in the 2K fraction. The numbers of cells present in the 0.3K and 2K fractions were quantitated using trypan blue staining as 1.35 × 10^5^ ± 3.69 × 10^4^ and 2.22 × 10^2^ ± 2.34 × 10^2^ (cells/1 mL OFs), respectively (Fig. 1c). Few cells were observed in the 10K and 160K fractions.

The presence of small particles (which were not visible by optical microscopy) in the 10K, 160K, and Sup fractions was confirmed by tapping mode AFM analysis in liquid, as previously described^20^. The obtained images revealed many round-shaped objects in both the 10K and the 160K fractions (Fig. S1a). The estimated average particle size in the 160K fraction was 52.7 ± 30.7 nm (Fig. S1b), which agreed well with a previous value for human sEVs prepared by density gradient centrifugation^21^. Unlike the 10K and 160K fractions, the Sup fraction contained only very tiny objects less than 35 nm in diameter (Fig. S1a); these objects resembled the AFM image of bovine serum albumin^22^, suggesting that few EVs were present in the Sup fraction.

The AFM images obtained in this study and in previous studies^20,21,23^ strongly indicated that the particles in the 10K and 160K fractions correspond to EVs. The detailed structures of these particles were revealed by TEM examination of ultra-thin sections from the 2K, 10K, and 160K pellets embedded in resin and positively stained to show lipid layer structures. As shown in Fig. 1d, the fractions contained particles of various sizes, and the 2K and 10K fractions had larger particles than those found in the 160K fraction. In agreement with studies on blood plasma^24^, seminal plasma^25,26^, cerebrospinal fluid^27^ and cultured cells^28^ using cryo-TEM, the OFs in the present study contained vesicles with multiple bilayers. An EV with a single bilayer was enriched in the 160K fraction, whereas the 2K fraction had EVs with multiple bilayers (white arrows), which sometimes contained the smaller vesicular-like entities within them (white arrows with asterisks) (Fig. 1d and Fig. S2a). The 2K and 10K fractions also contained many bacteria, which were characterized by their high density in TEM images (black arrows)^29^, whereas similar entities were absent from the 160K fraction. However, the 160K fractions contained small particles of high density (black arrowheads), which may represent bacteria-derived EVs or tiny bacteria (Fig. 1d and Fig. S2).

The results obtained from AFM and TEM observations indicated that the 10K and 160K fractions contained large numbers of vesicle particles. These particles were quantitatively studied by NTA analysis of fractions prepared from the OFs of 6 healthy volunteers (Table S1: Healthy control; HC 1–6). The 10K and 160K fractions contained 1.40 × 10^9^ ± 1.95 × 10^10^ and 3.84 × 10^10^ ± 2.88 × 10^10^ particles/mL, respectively, with diameters of 216.5 ± 17.5 nm and 132.8 ± 19.4 nm, respectively, (Fig. S3). The Sup fraction contained large numbers of small particles (2.83 × 10^10^ ± 2.02 × 10^10^ particles/mL with a diameter of 133.4 ± 21.0 nm) (Fig. S3). However, the non-vesicle-like structures observed in AFM (Fig. S1) and the higher total amount of proteins (70.5 ± 15.3%) in the Sup fraction than in the other fractions (Fig. S4) indicated that most of the particles present in the Sup fraction were non-EV particles, such as supramolecular protein complexes.

**Figure 2:**
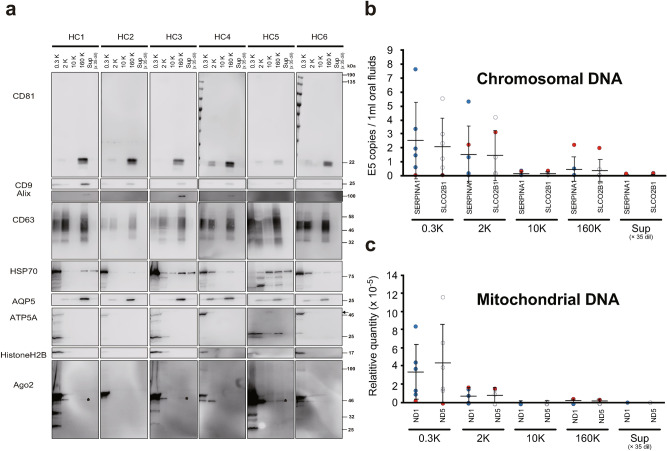
Distributions of protein markers, chromosomal DNA, and mitochondrial DNA in the pentapartite fractions of oral fluids (OFs) from 6 healthy volunteers. (**a**) Western blots of proteins markers for canonical exosomes (CD81, CD9, Alix, CD63 and HSP70), salivary gland (AQP5), mitochondria (ATP5A), chromatin (Histone H2B), and miRNA-associated protein (Ago2). Numbers on the right indicate molecular weight × 10^–3^. Black arrow shows the expected size of ATP5A. Asterisks in the 160K fractions of Ago2 are discussed in the text. Note that Sup was diluted 35-fold. The copy numbers of chromosomal DNA (**b**), and mitochondrial DNA (**c**), were determined by quantitative PCR. Target genes were SLCO2B1 and SERPINA1 for chromosomal DNA and ND1 and ND3 for mitochondrial DNA. The OFs were obtained from n = 6 healthy donors. Data are expressed as the mean ± standard deviation (SD). Dots indicate the mean value of the three measurements for each subject, while the mean values obtained from six subjects are represented as solid horizontal lines. Note that Sup was diluted 35-fold. The data obtained from HC45 are shown by red filled circles (see “Results” section).

The expression of protein markers was also analyzed by western blotting of the five fractions. A classical small EV (or exosome) marker, CD81, was enriched in the 160K fraction and was present in the 2K fraction (Fig. 2a). CD9 and Alix were also enriched in the 160K fraction, but their expression levels widely diverged among the study participants; in some specimens, the signals were very weak. The expression of aquaporin 5 (AQP5), although limited to the salivary gland in the oral space^30,31^, was also enriched in the 160K fraction (Fig. 2a) with less expression in the 2K and 10K fractions. CD63 and HSP70, two other proposed exosome markers, showed widely distributed expression among the five fractions. The presence of HSP70 in the Sup fraction was especially notable because the Sup fraction was diluted 35-fold during the experiments, indicating that large amounts of HSP70 exists in soluble form in the oral space. Similar distributions of CD81, CD63, and heat shock protein have been reported from conditioned medium for some cultured cell lines^32^.

### Distribution of chromosomal and mitochondrial DNAs

The presence of extracellular forms of RNAs, chromosomal DNA, and mitochondrial DNA have been reported in various body fluids, but whether they represent the cargos of certain types of EVs or whether they exist in non-vesicular forms remains to be established^33–35^. Recent studies have shown that stressed cells perform a regulated release of several types of sub-cellular components, including mitochondria^14,36–38^ and micronuclei^39^, which can serve as carriers of extracellular nucleic acids. Examination of the expression of Histone H2B (a core protein in nucleosomes) and ATP5A (a subunit of the mitochondrial ATP synthase) by western blotting confirmed that these proteins were mostly enriched in the 0.3K fraction, with small amounts in 2K fraction (Fig. 2a), suggesting an origin from chromosomes and mitochondria present in intact cells.

We also examined the distribution of extracellular nucleic acids by quantifying chromosomal DNA (SERPINA1; chromosome 14 and SLCO2B1; chromosome 11) and mitochondrial DNA (ND1 and ND3^40^) using real-time PCR, indicating that both chromosomal and mitochondrial DNAs were abundant in the 0.3K and 2K fractions. In the 0.3K, the fraction, the average copy number of chromosomal DNA (2.49 and 2.10 × 10^5^ copies/mL for SERPINA1 and SLCO2B1, respectively) agreed well with the cell numbers observed by trypan blue staining (Fig. 1c; 1.3 × 10^5^/mL of OFs. Note that each cell has two copies of chromosomal DNA). By contrast, in the 2K fraction, much more chromosomal DNA was detected than was expected from the number of cells present in the fraction. Optical microscopy observation of the 0.3K and 2K fractions after DAPI staining revealed the presence of nucleic acid-positive subcellular particles in the 0.3K fraction (Fig. S5a). Similarly, the amount of mitochondrial DNA present in the 2K fraction was higher than expected based on the numbers of cells present in the fraction. Chromosomal and mitochondrial DNA was also present in the 160K fraction; however, comparison of the copy numbers of DNA and particles indicated that only a minute fraction of the EVs carried DNA as a cargo, if DNA was associated with EV (Fig. S2 and Fig. 2). Interestingly, in one of six healthy volunteers (Healthy control case: HC5), the amount of DNA in the 2K fraction was far higher than in the 0.3K fraction (indicated by the red-filled circles in Fig. 2b,c). The western blots revealed a different distribution of ATP5A (mitochondrial marker) in HC5 than in other subjects, as the full-length protein was enriched in the 10K fraction rather than in the 0.3K and 2K fractions, while cleaved products were observed in the 0.3K, 2K, 10K, and 160K fractions (Fig. 2a). Thus, the distributions of nucleic acids, which may represent regulated extraordinary states of cell, varied widely among healthy individuals, even though the OFs were collected under similar conditions.

Bacteria have been also proposed to release their nucleic acids, along with bacterial EVs, into the extracellular space^41^. As shown above, TEM observations revealed various sizes of particles with high densities in the 2K, 10K, and 160K fractions (Fig. 1d and Fig. S2), and these most likely represent bacteria and bacteria-derived EVs. Assessment of the distribution of bacterial chromosomes in each fraction by semi-quantitative PCR targeting ribosomal RNA genes revealed the presence of DNA in the 0.3K and 2K fractions, and the increased cycles of PCR detected DNA in the 10K and 160K fractions (Fig. S5b). Further studies are needed to confirm whether these bacterial DNAs in the 10K and 160K fractions exist in a vesicle-free state or are associated with EVs.

### RNA molecules in the five fractions

RNases are widely present in tissues and body fluids, including OFs^42^. Development of an OF-based diagnostic system, especially one with a focus on OF RNA molecules, requires a careful setup of the analysis conditions to avoid post-sampling degradation of the RNA. For this purpose, we first tested the effects of adding proteinase K (PK) and 0.1% SDS, which have been reported to inactivate ribonucleases^43^, on the RNA profile of OFs. The assessments were based on the size distribution of the RNA profile determined with a bioanalyzer (Fig. S6a). Unexpectedly, the samples from two of the three healthy volunteers showed accelerated RNA degradation in response to the addition of PK and 0.1% SDS and a 12 h incubation at 37 °C. This degradation was characterized by the disappearance of the peaks of larger RNAs and increases in peaks of smaller RNAs in bioanalyzer profile for the 0.3K fraction (Fig. S6b). Therefore, in this study, RNA was prepared without addition of PK and SDS. The bioanalyzer results also indicated that the 0.3K, 2K, and 10K fractions contained an appreciable amount of ribosomal RNAs (rRNA) (Fig. S6b). We determined the origin of these rRNAs (human body or oral bacteria) by running RNA prepared from OF along with control human cell RNA and bacterial RNA. This experiment indicated that most of the rRNAs in the OFs were of bacterial origin (Fig. S6c–h).

The average amounts of RNA isolated were estimated by the Bioanalyzer as 64.2 ± 53.1, 37.6 ± 39.8, 0.9 ± 1.1, 3.4 ± 3.7, and 0.3 ± 0.4 ng/mL for the 0.3K, 2K, 10K, 160K, and Sup fractions, respectively, with large standard deviations (data not shown). The numbers of particles also diverged between individuals (Fig. S3a), and moderate correlations were observed between the amounts of RNA and the numbers of particles in the 10K, 160K, and Sup fractions, implying an association between the RNA and the particles (Fig. S7).

In our previous study, we showed that sEV-enriched fractions prepared by density gradient ultracentrifugation contained miRNAs^21^. However, this observation did not exclude the possibility that other fractions (i.e., the fractions containing larger EVs) also contained miRNAs. We determined the distributions of the miRNAs among the five fractions by digital PCR-based quantification for four miRNAs confirmed to be present in OFs in our previous study^21^. At least for the miRNAs investigated, the richest sources were the 0.3K and 2K fractions that contained the larger EVs, but not the 160K fraction (Fig. S8). Argonaute2 (Ago2) has been proposed to associate with miRNA in extracellular space^44^. Jeppesen et al*.* have shown that the protein was present both in larger and smaller EV crude fractions^33^. In our western blots, Ago2 was present both in the 2K and 160K fractions from subjects HC1, HC3, and HC5 (Fig. 2a), supporting the observation of Jeppesen et al^33^. The higher expression of Ago2 and miRNAs in HC5 (Fig. 2a and Fig. S8) also suggests the possible role of Ago2 in the secretion of miRNAs into extracellular space. By contrast, the expression patterns of CD81, CD9, CD63, and AQP5 or particle numbers in the 160K fraction (Fig. 2a and Fig. S3) were not correlated with any of the tested miRNAs, suggesting that non-canonical sEVs (or non-EV particles) carry miRNA in the 160K fraction. Notably, Murillo et al. have reported that extracellular RNA in saliva is correlated with high density lipoprotein (HDL)^45^. We also looked for a correlation between miRNA and HDL by investigating the expression of ApoA1, an HDL marker, in subject HC4 (a low miRNA expression sample) and HC5 (a high miRNA expression sample), and we found that ApoA1 expression was enriched in HC5 (Fig. S9), supporting the possibility of HDL as miRNA carrier in the 160K fraction.

### Comparisons of OFs from patients with OSCC and from healthy volunteers

Assembling the vast quantity of clinical data needed to establish a new diagnostic system requires that the patient’s OFs undergo freezing for storage and a thawing step for analysis. Either or both of these steps could adversely affect the results of analyses by decreasing the number of particles or by reducing the western blot signals^46^. In the experiments already mentioned (Figs. 1, 2), we used fresh OFs that had not been frozen and thawed. We therefore evaluated the effects of the freezing/thawing cycle on the properties of OFs by first comparing the numbers of particles in the 10K, 160K, and Sup fractions prepared from healthy volunteers. The calculated particle numbers in these fractions were not affected by the storage condition of the OFs (Fig. S10). Furthermore, the addition of a freezing step did not dramatically alter the expression patterns observed by western blotting, indicating that a single freezing/thawing cycle is compatible with high-throughput analyses of clinical OF samples.

Based on these observations, we adopted a storage step at − 80 °C before fractionation as a standard protocol. We analyzed OFs from both patients and controls under identical conditions by collecting OFs from five healthy volunteers (HC7 to HC12) and six patients with OSCC (OSCC1 to OSCC6). The patients with OSCC selected in this study included three with squamous cell carcinoma of the tongue and three with squamous cell carcinoma of the gum; all were at stage III and IV (Table S1). The 12 OF specimens were separated into five fractions and studied by silver staining and NTA particle analyses. The silver staining revealed no significant differences between healthy controls and patients with OSCC (Fig. S11).

**Figure 3:**
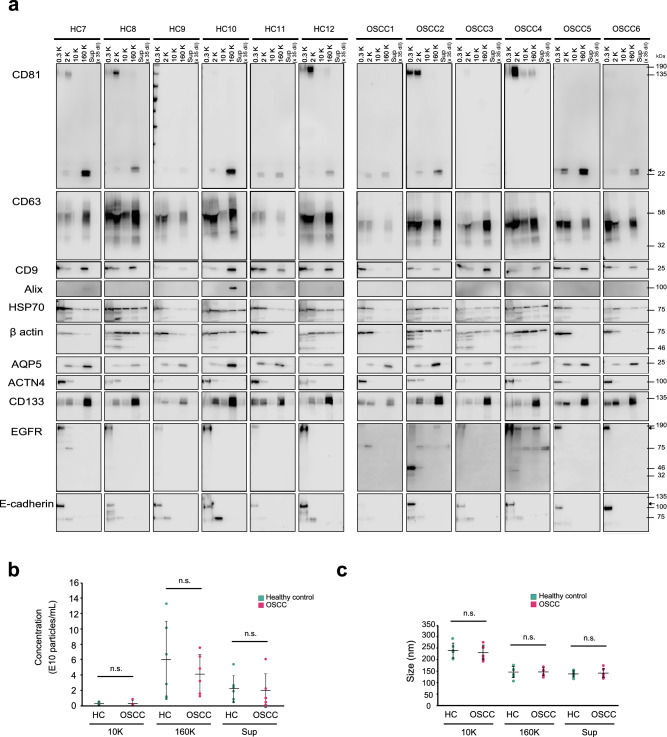
Comparative analyses of pentapartite oral fluid (OF) fractions between healthy volunteers and patients with OSCC. (**a**) Western blot analyses of pentapartite fractions from six healthy subjects (HC7 to HC12) and six patients with OSCC (OSCC1 to OSCC6). Membranes were probed with antibodies against CD81, CD63, CD9, Alix, Hsp70, β-actin, AQP5, ACTN4, CD133, EGFR, and E-cadherin. Numbers on the right indicate molecular weight (MW) × 10^–3^ of for MW markers. Black arrows show the reported sizes of the proteins. Nanoparticle tracking analyses (NTA) of particles contained in the 10K, 160K, and Sup fractions derived from healthy controls and patients with OSCC. Particle concentrations (**b**), and mode diameters (**c**), are indicated by green and red dots, respectively. The mean value of each group is represented as a solid horizontal line. The two-tailed unpaired *t-*test was used to evaluate statistical significance. Statistical significance was defined as p < 0.05. *n.s.* not significant.

Some studies have reported that the numbers or sizes of EVs had characteristic alterations in cancer patients^23,47^; however, our analyses did not reveal any significant differences in the numbers and sizes of particles in the 10K, 160K, and Sup fractions of the heathy controls and patients with OSCC (Fig. 3b,c). The western blots also showed no obvious differences in the expression patterns of CD63, CD9, CD133, and AQP5 in the five OF fractions from healthy donors and patients with OSCC (Fig. 3a). The protein α-actinin-4 (ACTN4, an actinin-binding cytoskeleton protein) has also been proposed as a tissue marker of OSCC^48^. In the western blots, this protein was found in the 2K fraction (Fig. 3a), which agreed well with previous reports indicating that α-actinin-4 is associated with larger EV^32,33^. The expression of this protein in the 2K fraction did not differ markedly between the healthy volunteers and the OSCC patients.

The EGFR signaling pathway is activated in many tumors through various mechanisms^49^, and some cancer cell lines have been reported to release EGFR-containing exosomes^33,50–53^. Western blot investigation of EGFR expression in the five OF fractions revealed the presence of the full-length EGFR mostly in the 0.3K fraction (Fig. 3a) in both healthy and OSCC samples, which may reflect expression of EGFR in oral epithelial cells^54^. Interestingly, the full-length EGFR was also observed in the 160K fractions from two patients with OSCC: OSCC2 and OSCC4 (Fig. 3a). This finding agreed well with the reported release of EGFR-positive exosomes from cancer cells^33,50,52,53^. These two patients were diagnosed as having cervical lymph node metastasis (Table S1), thereby supporting the proposed role of the EGFR^+^ EVs in cancer progression^53^.

EGFR has been also known to be present as shorter derivatives produced by genetic or enzymatic modifications^50,55–58^. Some of these derivatives are reportedly released into the extracellular space^50,55,59^, and we also observed shorter forms of EGFR with apparent molecular weights of 75K and 45K (Fig. 3a). The monoclonal antibody we used recognizes the C-terminal region (the antigen was the peptide around 1068 position of EGFR and it can recognize both phosphorylated and non-phosphorylated EGFR). A further examination of the shorter derivatives by western blotting with a monoclonal antibody recognizing the N-terminal region (aa 30–198) of EGFR (Fig. S12a) revealed that the shorter derivatives were categorized into three groups denoted in this paper as EGFR^C75kDa^, EGFR^C45kDa^ and EGFR^N45kDa^ (shown as red, cyan, and green arrowheads in Fig. S12a). The EGFR^C75kDa^ was present in the 2K fraction from two healthy donors and from three patients with OSCC and was recognized only by the C-terminal specific antibody (Fig. S12; red arrowheads). The intracellular domain of EGFR is not heavily glycosylated; therefore, the EGFR^C75kDa^ must be the derivative that lacks the whole extracellular domain but retains the transmembrane segment that allows anchorage to vesicles (Fig. S12b). EGFR^C75kDa^ was not observed in the 0.3K fraction, so enzymatic shedding during EV formation may be the underlying mechanism that produces this form. Examination of the EGFR^C75kDa^ from healthy individuals suggested this form is not associated with OSCC progression. However, the OSCC2 and OSCC4 samples contained the EGFR^C75kDa^ in fractions other than 2K. Its presence in the Sup fraction is intriguing because the Sup fraction was diluted 35-fold for western blot experiments, indicating that a large portion of EGFR^C75kDa^ was present as a soluble form in subjects OSCC2 and OSCC4.

EGFR^C45kDa^ was also recognized by the C-terminal-specific antibody but not by the N-terminal antibody (Fig. S12; cyan arrowheads). This form was not observed in healthy individuals but was present in OSCC2 and OSCC4. In contrast to the EGFR^C75kDa^, this form was present in the 0.3K fraction of OSCC2 in addition to the 2K fraction, suggesting that EGFR^C75kDa^ and the EGFR^C45kDa^ were produced under different cellular conditions. Considering its apparent size, the EGFR^C45kDa^ may lack the transmembrane segment, making it a soluble cytoplasmic protein, although the possibility cannot be excluded that it was produced from alternative splicing and retains the ability to bind to membranes. The distinct partitioning of EGFR^C45kDa^ (0.3K and 2K) and the slight differences in apparent size (45 kDa and 35 kDa in OSCC2 and OSCC4, respectively) may further differentiate this subclass of EGFR derivatives.

The third major derivative, EGFR^N45kDa^ was only detected with the N-terminal antibody (Fig. S12; green arrowheads), indicating that it corresponds to the extracellular domain of EGFR. It was observed only in the 0.3K fraction from two healthy individuals, HC3 and HC4. Because it did not seem to be secreted into the extracellular space, this form may be associated with the process of EGFR degradation within cells. The exosomes released from HaCaT cells reportedly contain the full-length EGFR as well as the C-terminal 55 kDa fragment and 150 kDa and 100 kDa N-terminal EGFR fragments, which have been proposed to be produced during proteolytic processing^50^. A focus on the 0.3K fraction revealed the C-terminal specific form. Similarly, MDA-MB-468 cells released a 110 kDa N-terminal EGFR fragment into the extracellular space by metalloprotease processing^55^. Because these truncated EGFR forms have different molecular sizes and localizations, the processing of EGFR should involve various factors to produce various isoforms.

Western blotting using the N-terminal specific and the C-terminal-specific antibodies also differentiated the whole-length EGFR into two subtypes. In the 0.3K fraction, the C-terminal specific antibody detected the apparently full-length EGFR from all samples except OSCC1, which had low expression of EGFR. By contrast, the N-terminal specific antibody showed a signal only in HC6, OSCC2, and OSCC4 samples at around 160K, suggesting that the eight other samples had lost the N-terminal epitope for DAK-H1-WT. In the 160K fraction, the C-terminal-specific anti-EGFR antibody detected the apparently full-length EGFR (shown by asterisks in Fig. S12b) both from OSCC2 and from OSCC4 (shown by asterisks in Fig. S12b), whereas only the one in OSCC4 was detected by the N-terminal specific antibody. A further complicating observation was detection of the apparently full-length EGFR in the 2K fraction from HC6 and OSCC2 by the N-terminal specific antibody but not by the C-terminal antibody. The 140 kDa EGFRvIII variant, which lacks exons 2–7, is frequently observed in some cancers; however, this form has been reported as atypical in OSCC^60^.

Thus, various forms of EGFR were found among the five fractions of OFs, and these should reflect the proteolytic degradation of the protein^50^, as well as any genetic modifications (genome rearrangement and alternative splicing)^61^. EGFR is not a particular case that produces various derivatives, but many proteins, and especially membrane proteins, have the capability to produce shorter variants in a programmed manner^62^. One example is E-cadherin, and the extracellular domain of this protein (80 kDa, ECAD^80kDa^) is cleaved off by MMPs^63^ and released as a soluble factor into the blood stream in some cancers, including gastric and bladder cancers^64,65^. Our western blots using the N-terminal specific antibody to E-cadherin also detected a shorter variant of E-cadherin, with an approximate molecular weight of 80 kDa (Fig. 3a). Interestingly, in the case of OFs, this ECAD^80kDa^ was mostly enriched in the 2K fraction, suggesting the possibility that the ECAD^80kDa^ behaves as soluble entity that loads onto large EVs.

The enrichment of EGFR^C75kDa^ and ECAD^80kDa^ in the 2K fraction excluded the possibility that the signals in the 2K fraction were derived from contaminating cells from the 0.3K fraction. The TEM observations revealed that some EVs with double bilayers contained the smaller EVs within them (Fig. 1c and Fig. S4b). Therefore, we examined the possibility that autophagosome-related large organelles^33,66^ were enriched in that fraction. We addressed this possibility by examining the expression of LC3 by western blotting. LC3 has important roles in autophagosome generation and fusion with lysosomes, and its expression in tumors has been proposed to serve as prognostic marker of OSCC^67^. LC3 was present in two forms in western blots: LC3-I with an apparent size of 17 kDa and LC3-II, which is the phosphatidylethanolamine (PE) conjugated form of LC3-I. In SDS-PAGE, LC3-II runs faster than LC3-I. LC3-II is present in autophagosomes^66,68^. The results from the five OF fractions have revealed a clear enrichment of LC3-II in the 2K fraction in some samples, supporting the possibility that autophagosome-related EVs are recovered in the 2K fraction (Fig. S13). In some cases, LC3-I and/or LC3-II were also present in other fractions (from HC7, HC10, HC8, and OSCC4), indicating that smaller autophagosome-related EVs may be formed under certain circumstances. Unexpectedly, the western blots of OFs also revealed the presence of multiple ladder-like signals in most cases (Fig. S13). Previous studies have reported that LC3-I forms a stable covalent complex with Atg3 and Atg7 and associates with other proteins, such as DOR, lamin B1, MAP1B, tubulin, and 40S ribosomal proteins^69–72^. These ladder-like bands in the 2K and 160K fractions could represent the various proteins to which LC3 was covalently linked. Notably, in OSCC 2 and OSCC4, which were characterized by multiple EGFR fragments, the ladder-like bands were particularly evident (Fig. S12 and Fig. S13).

**Figure 4:**
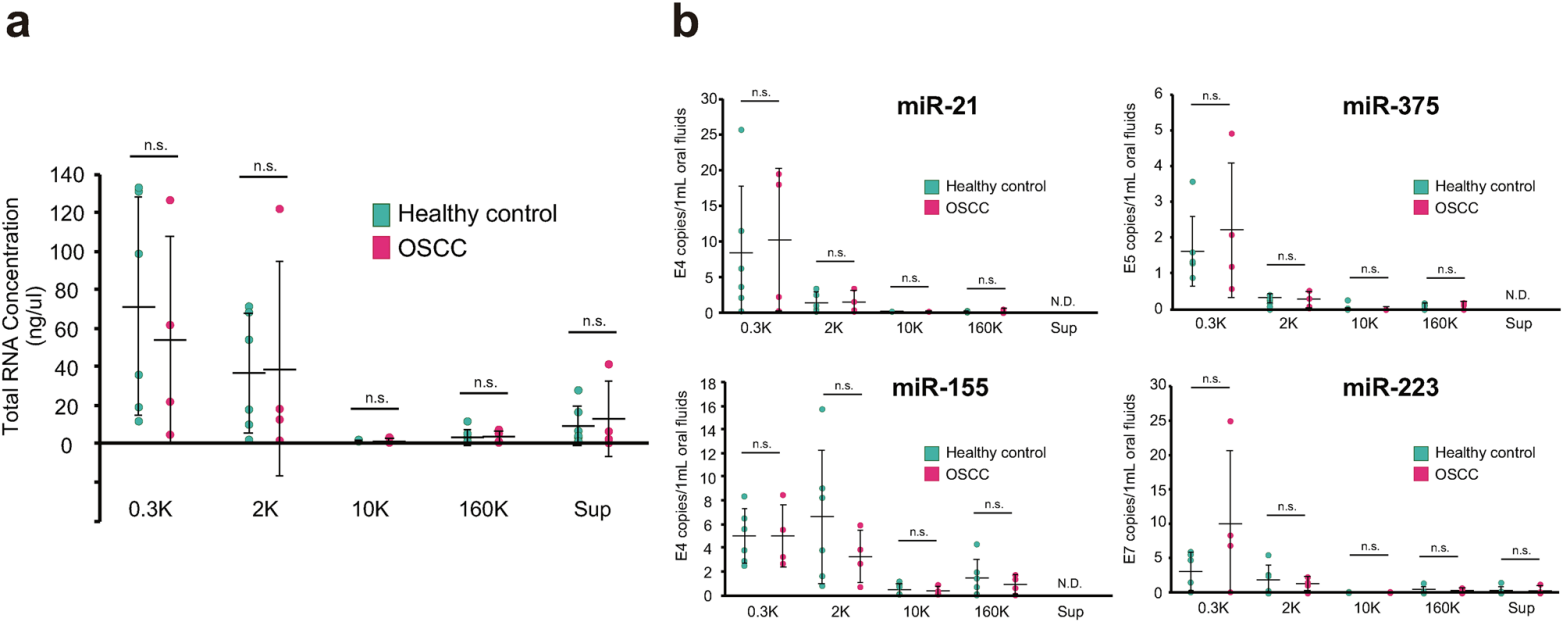
Distribution of RNA molecules among pentapartite fractions from oral fluids (OFs). (**a**) Total RNA concentration in each fraction from 6 healthy volunteers (green dots) and 4 patients with OSCC (red dots) estimated by Bioanalyzer. Each dot represents the mean value obtained from three measurements. (**b**) Copy numbers of miR-21, miR-375, miR-155, and miR-223 in each fraction were determined using chip-based Quant Studio 3D digital PCR. Green and pink dots indicate the mean values of the three measurements in 6 healthy volunteers and 4 patients with OSCC, respectively. The mean value of groups is represented as solid horizontal lines. The two-tailed unpaired t-test was used to evaluate statistical significance. *n.s.* not significance, p > 0.05.

We also investigated the amounts of total RNAs and the expression of some miRNAs (miR-223, miR-21, miR-155, and miR-375) in the five fractions, but found no pronounced differences between patients with OSCC and healthy donors in terms of total RNA concentration (Fig. 4). The four tested miRNAs have been proposed as biomarkers in tissue^73,74^ or blood^75,76^ samples, but our data suggested that information obtained from other body fluids cannot necessarily be applied to OFs.

## Discussion

**Figure 5:**
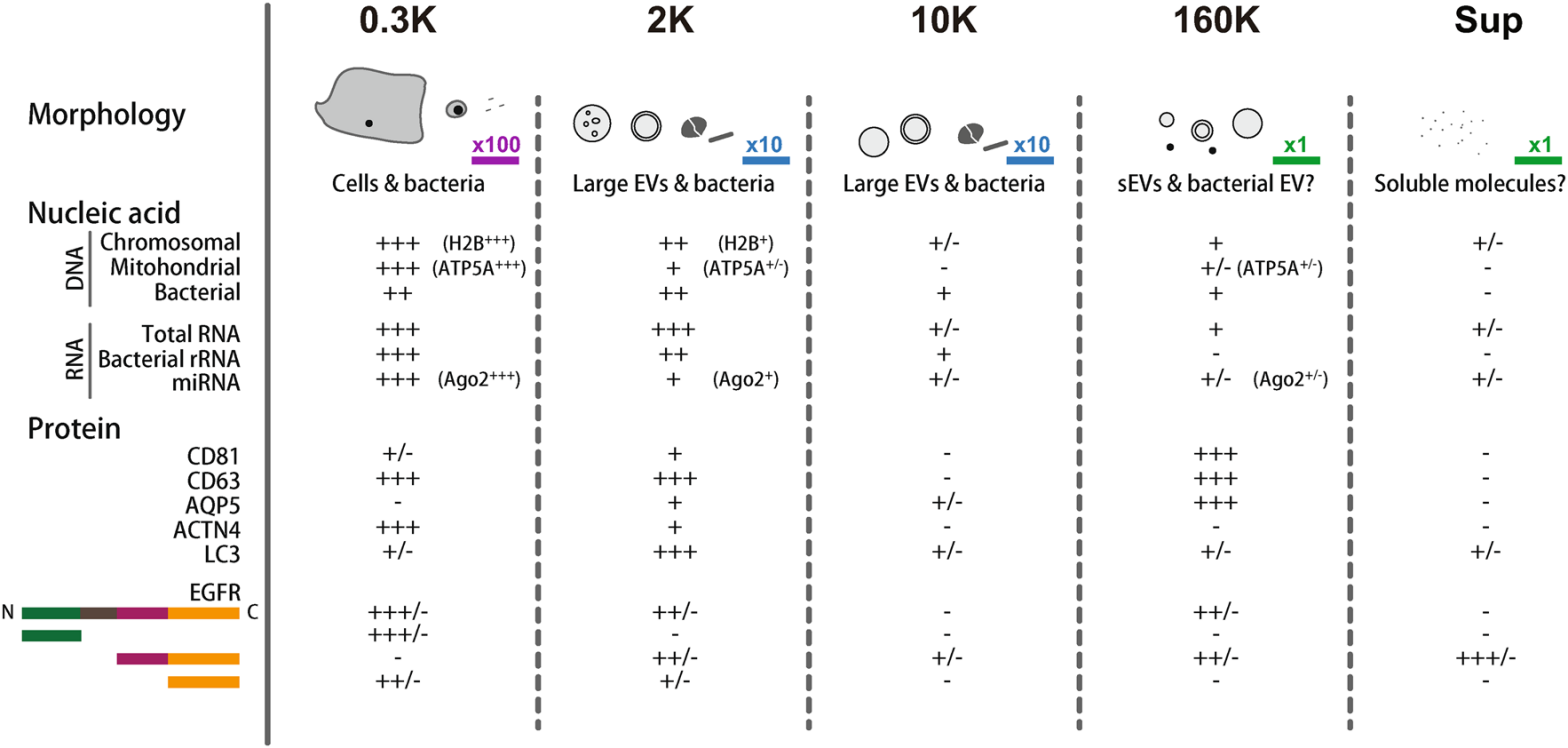
Summary of pentapartite fractionation of human oral fluids. At the top, schematic illustrations of particles observed are shown. The scale bars labeled with × 100, × 10, and × 1 correspond to 20 μm, 2 μm, and 200 nm, respectively. Representative protein markers are shown. “+/−” represents some specimens gave positive signals. Details are described in the “Results” section.

The purpose of this study was to obtain a complete picture of the particulate matter present in human OFs (Fig. 5). Since their discovery as a carrier for nucleic acids cargo in 2007^77^, exosomes have been attracting a great deal of attention in basic and applied biology and have already established a position as important messengers that package and carry various molecules between cells. A focus on the small particles present in body fluids is therefore expected to generate more accurate diagnostic information than is possible by analyzing the body fluids alone. However, the exosome is not only the vesicle that is released from cells; different types of vesicles are generated from cells by various mechanisms and these vesicles are collectively called “EVs”^46^. Multivesicular body-mediated secretion, direct budding from plasma membrane, exfoliation from cellular protruding structures, autophagosome mediated generation, and regulated cell death-dependent pathways are thought to contribute to the generation of different forms of EVs^9,10^. There is also no reason to believe that the multivesicular body-mediated sEV (often called an exosome, although the term exosome has been differently defined by several groups^8^) is the only vesicle that carries clinically important information. Indeed, larger vesicles could carry more important information for the diagnosis of certain diseases^12–18^. Given these possibilities, we have to first determine which type of EV (or even non-EV moiety) should be the focus for a particular diagnostic purpose.

To provide guidelines for exploring the OF-based diagnostic system, our aim in this study was to reveal a complete picture of the particulate matter in OFs based on their sizes. For this purpose, we separated OFs into five fractions using differential centrifugation (Fig. 1a). The 0.3K fraction contained huge particles that included cells and bacteria, whereas the 2K fraction contained large particles of sub-cellular size. The 10K fraction has been often used as a source of microvesicles or medium size EVs. The 160K fraction has been widely used as a crude exosome or sEV fraction, from which more enriched sEVs are prepared by appropriate methods. The final supernatant (Sup) was also analyzed here for particle properties and biomarker distributions.

The differential centrifugation method differentiates particles based on their sizes. Reflecting this principle, the 0.3K fraction contained many epithelial cells and immunocytes, along with oral bacteria. Under the conditions used, an average of 1.3 × 10^5^ cells was present per mL of OFs. The 2K fraction contained few intact cells; instead, it contained subcellular-sized particles viewable by optical microscopy (Fig. 1b). TEM observations of the 2K fraction revealed the presence of large EVs with double bilayers that often contained smaller single bilayer vesicle within them (Fig. 1d and Fig. S2). Western blots revealed that the 2K fraction contained the autophagosome-related LC-3 protein (Fig. S13), implying that autophagosome-related EVs were enriched in the fraction. We employed a sonication treatment to reduce the viscosity of the OFs, so this raised the possibility that the 2K fraction was an artificial fraction resulting from mechanical breakage of intact cells from the 0.3K fraction. However, some protein markers were uniquely expressed in the 2K fraction, indicating that the 2K fraction contains idiosyncratic entities.

CD9, CD63, CD81, and Alix have been often used as exosome markers^46^. However, as many studies have already shown, these are not uniquely expressed in sEVs or in MVB-mediated sEVs^32^. This also holds for human OFs. CD63 and CD9 were expressed in the 160K fraction, as well as in the 0.3K and 2K fractions, and the expression of Alix diverged between specimens. CD81 was mostly enriched in the 160K fraction, but it was also found in the 2K fraction in amounts that were not negligible. In addition, CD81 formed an apparently huge complex in the 2K fraction according to western blots (Fig. 3), but the origin of this complex is not yet known. Along with CD81, AQP5 was mostly enriched in the 160K fraction, with some found in the 2K fraction. AQP5 is believed to be exclusively expressed in the salivary gland in the oral cavity^30,31^, and the absence of the signal of AQP5 in the 0.3K fraction confirmed that epithelial cells do not express this protein. Our observations suggested the 2K and the 160K fractions a possible source for liquid biopsy for salivary cancer to replace current invasive fine needle aspiration cytology (FNAC) methods^78^. For instance, immunoactivity-based capture using the anti-AQP5 antibody will enable us to concentrate salivary gland-derived EVs, which should carry diagnostic molecules such as fusion transcripts^79^. Alternatively, sandwich ELISA, in which the anti-AQP5 antibody will be combined with other appropriate antibodies, will be explored as a simple diagnostic method.

Exosomes carrying RNA molecules and DNA molecules have been attracting great attention as diagnostic markers for various diseases. However, as shown in this work, OFs contain most of their nucleic acid molecules in the 0.3K and 2K fractions, which may represent epithelial cell-origin nucleic acids. When the sEVs carrying DNA or RNA are used for OF-based diagnosis, the sEVs should be carefully separated to avoid any carryover of nucleic acids from the 0.3K and 2K fractions.

Recent studies have also reported the secretion of mitochondria into the extracellular space^14,36–38^. Our data indicated that mitochondrial DNA was distributed among the pentapartite fractions in a manner similar to that of chromosomal DNA (Fig. 2b). However, no intact mitochondrial structures were not observed in TEM images, suggesting that mitochondrial DNAs were secreted as particular derivative forms of mitochondria.

Many membrane receptors, including EGFR or E-cadherin, are known to produce various shorter derivatives by different mechanisms, including enzymatic digestion, chromosomal alteration, and mRNA splicing, and these have been proposed to be associated with the progression of the cancers^50,55–58,64,65^. Some derivatives were found in the 160K fraction and others were present in the 2K fraction. It is worth noting that previous studies have indicated that EGFR is mostly associated with non-canonical exosomes^33^ and larger EVs^51^. A plausible membrane-bound form of EGFR derivatives was highly enriched in the Sup fraction of OFs from some OSCCC patients. The multiform nature of these truncated derivatives complicates the elucidation of their biological importance; however, our pentapartite analysis approach should allow the development of a deeper understanding of these derivatives and provide novel applications of them in diagnosis.

## Materials and methods

### Oral fluid collection

Oral fluid samples were obtained from healthy volunteers and patients with OSCC with their informed consent. Ethical approval was obtained from the Tokyo Dental College of Ethics Committee (approval number: 1-16-46R, 702) and the Japanese Foundation for Cancer Research (approval number: JFCR 2013-1112). This study was conducted according to the principles of the Declaration of Helsinki, and the informed consent was obtained from all participants. Donors were negative for a history of HIV, autoimmune disorders, hepatitis, and malignancy. The donors were asked to avoid eating, drinking, smoking, or oral hygiene procedures for at least 1 h before sampling. At 9 AM, the donors were directed to expectorate 5 mL of unstimulated OF into a 50 mL Cellstar tube (227270, Greiner Bio-One, Kremsmünster, Austria). The sample was placed on ice upon collection and then immediately stored at − 80 °C until analysis in subsequent experiments. Basic information, including age, sex, and tumor location, for all patients and the healthy controls is summarized in Table S1.

### Fractionation of oral fluids by differential centrifugation

A 5 mL volume of OF was mixed with 1 U/µL RiboLock RNase Inhibitor (EO0381, Thermo Fischer Scientific, MA, USA) and sonicated in a closed-type sonication system (UCD-200T, Biorupter, Diagenode Inc., NJ, USA) for a run time of 10 min, comprising 30 s pulses at 1 min intervals at medium power, as previously described^21^. The OFs were centrifuged at 300*g* for 10 min (H-3R, KOKUSAN, Saitama, Japan) to obtain a 0.3K pellet. The supernatant was spun again at 2000*g* for 10 min (H-3R, KOKUSAN) to obtain a 2K pellet. The supernatant was made up to 30 mL with phosphate buffered saline (PBS, 137 mM NaCl, 2.68 mM KCl, 8.10 mM Na_2_HPO_4_, 1.47 mM KH_2_PO_4_, pH 7.4) in Ultra-Clear Centrifuge Tubes (364772, Beckman Coulter, CA, USA) and centrifuged at 10,000*g* (L-90K with SW32Ti rotor, Beckman Coulter) for 30 min to obtain a 10K pellet. The supernatant was then centrifuged at 160,000*g* (L-90K with SW32Ti rotor) for 70 min to obtain a 160K pellet. All centrifugations were done at 4 °C. The pellets were not washed to prevent the loss of EV. Instead, the pellets were resuspended in 500 µL of the supernatants left over during collection of the supernatants into new tubes. Therefore, each 0.3K, 2K, 10K, and 160K fraction had a 10% carryover of its supernatant.

### Papanicolaou staining

Samples of each fraction were smeared on glass slides (Muto Pure Chemicals, 5116-20F, Tokyo, Japan) using a wedge method and stained with Papanicolaou solution^19^. The images were obtained either with a BX50F light microscope (Olympus, Tokyo, Japan) using 10 × and 20 × objective lenses (for the 0.3K, 2K, 10K, and 160K fractions) or with a Keyence BZ-X700 light microscope (Keyence, Osaka, Japan) using 20 × and 40 × objective lenses (for the OFs).

### Phase-contrast microscopy and fluorescence microscopy

Samples (20 µL) were transferred to collagen type I Cellware 8-well culture slides (354630, BD Biosciences, NJ, USA) and incubated at room temperature for 1 h. After removing the supernatant, the pellets were fixed in 4% paraformaldehyde (26126-25, Nacalai Tesque) at room temperature for 10 min, followed by washing with PBS, mounting on culture slides treated with ProLong Diamond Antifade Mountant, and staining with 4,6-diamidino-2-phenylindole (DAPI; p36962, Life Technologies, MA, USA). Phase-contrast and fluorescence images were obtained with the Keyence BZ-X700 microscope. The cells were stained with trypan blue (T8154, Sigma-Aldrich, MO, USA), and cell numbers were counted using a cell counter (BMS-OCC01, Bio Medical Science, Tokyo, Japan).

### Transmission electron microscopy

The EV pellets collected at the bottom of tubes were directly fixed with 100 μL of modified Karnovsky’s fixative solution^80^ (2.0% paraformaldehyde and 2.5% glutaraldehyde in 0.1 M cacodylate buffer, pH 7.2) for 1 h at 4 °C. After fixation, the pellets were carefully recovered with a spatula and embedded in LR White resin (Agar Scientific, Stansted, Essex, LDN, UK). Ultra-thin sections were prepared with an Ultracut UCT microtome (S9329, Leica, S9329, Wetzlar, Germany) and the specimens were stained with 6% uranyl acetate (Wako, Tokyo, Japan) and 3% lead citrate (Wako) on formvar-coated (Nissin EM, Tokyo, Japan) nickel grids (S-300 square mash, Gilder, Grantham, UK). Transmission electron microscopy (TEM) images were obtained with a H-7650 instrument (Hitachi, Co., Tokyo, Japan).

### Nanoparticle tracking analysis

The numbers and sizes of particles in each fraction were estimated by nanoparticle tracking analyses (NTA) using the NanoSight LM10 system (Malvern Instruments, Worcestershire, UK), as previously describe^20,81^. In brief, silica beads with a diameter of 150 nm (24320, Polysciences, PA, USA) were used as a calibration for the 10K fraction, whereas 100 nm diameter beads (24041, Polysciences) were used for the 160K fraction and supernatant. The camera level (CL) and detection threshold (DT) were set at values of CL 12/DT 10 and CL 14/DT 4. The samples were diluted with PBS filtered through a 0.1 µm syringe filter (6789-1301, GE Healthcare UK Ltd., Buckinghamshire, UK) to reach the desired concentration between 2 × 10^8^ and 1 × 10^9^ particles/mL. This concentration was based on silica beads, with a known concentration to correct for unevenness. For each sample, 30 s captures per sample were recorded, and each measurement was independently performed five times. NTA software version 2.3 (Malvern Instruments) was used for data analyses. An average histogram was plotted from the data of five measurements.

### Western blot analyses

The following primary and secondary antibodies were used for western blot analyses with the indicated dilution rates: Rabbit anti-ACTN4 (GTX101669, Gene Tex, CA, USA; 1:1000), Rabbit anti-Ago2 (ab32381, Abcam; 1:500), Mouse anti-Alix (634502, BioLegend, CA, USA; 1:1000), Rabbit anti-Aquaporin 5 (ab134687, Abcam, Cambridge, UK; 1:500), Mouse anti-ATP5A (ab14748, Abcam; 1:1000), Mouse anti-β-actin (A1978, Sigma-Aldrich, MO, USA; 1:1000), Mouse anti-CD9 (SHI-EXO-M01, Cosmobio; 1:1000), Mouse anti-CD63 (SHI-EXO-M02, Cosmobio; 1:1000), Mouse anti-CD81 (SHI-EXO-M03, Cosmobio; 1:1000), Mouse anti-CD133 (PAB12663, Abnova Corp, Taipei city, Taiwan; 1:1000), Mouse anti-E-cadherin (ab1416, Abcam; 1:500), Rabbit anti-EGFR (EP38Y, ab52894, Abcam;1:1000), Mouse anti-EGFR/DAK-H1-WT (M7298, Dako, Denmark; 1:1000), Rabbit anti-Histone H2B (EP957Y, Abcam; 1:1000), and Rabbit anti-HSP70 (EXOAB-HSP70A-1, System Biosciences, CA, USA; 1:1000). Secondary antibodies coupled to horseradish peroxidase were obtained as a Goat anti-Rabbit IgG (H + L)-HRP Conjugate (170-6515, Bio-Rad, CA, USA; 1:2000), Goat anti-Mouse IgG (H + L)-HRP Conjugate (170-6516, Bio-Rad; 1:2000), and Rabbit anti-Goat IgG (H + L)-HRP Conjugate (Jackson Immuno Research Laboratories, PA, USA; 1:2000).

For sodium dodecyl sulfate polyacrylamide gel electrophoresis (SDS-PAGE), 18 μL samples of each fraction were incubated with 6 μL of reducing sample buffer (250 mM Tris HCl (pH 6.8), 20% sucrose, 8% SDS, 5% 2-mercaptoethanol, 0.008% bromophenol blue) or non-reducing sample buffer (as above but without 2-mercaptoethanol) and boiled at 90 °C for 10 min. Proteins were separated by 7.5–15% SDS-PAGE (Extra PAGE One Precast Gel, Nacalai Tesque, Inc., Kyoto, Japan) in SDS running buffer (25 mM Tris, 191 mM glycine, 0.1% SDS) at a constant 1000 V, 40 mA for 40 min. Proteins were electro-transferred onto 10 cm × 10 cm nitrocellulose membranes (IB201002, Invitrogen, CA, USA) using the iBlot dry blotting system (Invitrogen)^21^. Nonspecific binding sites were blocked by incubating the membrane in 10 mL of Blocking One (03953-95, Nacalai Tesque, Inc.) for 1 h, before washing 3 times for 5 min with 10 mL of Tris-buffered saline-Tween [TBS-T, 10 mM Tris–HCl, 150 mM NaCl, 0.02% Tween-20 (P1379, Sigma-Aldrich)]. Membranes were probed with primary antibody overnight in 10 mL of Can Get Signal Solution 1 (NKB-101, Toyobo Co, Ltd., Osaka, Japan), followed by incubation with horseradish peroxidase-linked secondary antibody in 10 mL of CanGet Signal Solution 2 for 30 min in darkness. All antibody incubations were carried out using gentle orbital shaking. Membranes were washed 3 times with 10 mL TBS-T for 5 min after each incubation step^21^. Primary antibodies were detected using ECL (A-8511, C-9008, Sigma-Aldrich) with 3% H_2_O_2_ and visualized with an Odyssey Fc Imaging System (2800-00, LI-COR, Inc., NE, USA).

### DNA analyses

Samples (100 µL) were incubated with 0.1% SDS for 30 min at 25 °C, followed by treatment with. 100 μg/mL Proteinase K Solution (162-22751, Wako Pure Chemical Industries, Ltd., Osaka, Japan) for 12 h at 37 °C. An equal volume of UltraPure Buffer-Saturated Phenol (15513-039, Invitrogen) was then added to all samples. After vortexing, the mixture was centrifuged at 2000*g* for 10 min (Centrifuge 5415R, Eppendorf, Hamburg, Germany) at room temperature. The resulting supernatant was transferred to a new tube and vortexed with 100 µL of phenol (15513-039, Invitrogen):chloroform (08402-55, Nacalai Tesque):isopropyl alcohol (166-04836, Wako Pure Chemical Industries) (25:24:1, v/v). After centrifugation at 2000*g* for 10 min (Centrifuge 5415R, Eppendorf), the upper aqueous phase was transferred to a new tube and vortexed with 100 µL of chloroform:isoamyl alcohol (24:1, v/v). The mixtures were then centrifuged at 2000*g* for 10 min (Centrifuge 5415R, Eppendorf) and the upper aqueous phase was transferred to a new tube. Ethanol was added to condense and precipitate the DNA. Finally, the DNA samples were dissolved in 50 µL Tris–EDTA buffer (T0221, Teknova Inc., CA, USA).

Chromosomal DNA and mitochondrial DNA in each fraction were quantified using the Human Mitochondrial DNA (mtDNA) Monitoring Primer Set (7246, TaKaRa, Shiga, Japan), according to the manufacturer’s protocol. Briefly, the relative number of copies of human mitochondrial DNA were quantified by real-time PCR with SYBR Premix EX Taq II (Tli RNase H Plus) (RR820SA/B, TaKaRa). Human genomic DNA (636401, Clontech, CA, USA) was used as a standard. PCR was carried out in triplicate in a 20 µL reaction volume. Data were analyzed using the Applied Biosystems 7500 Fast Real Time PCR Systems. Quantification of copy numbers for mitochondrial DNA (ND1) were performed by using digital PCR, as previously described^40^.

### RNA analyses

Total RNA was extracted using TRIzol Reagent (15596-018, Life Technologies), as previously described^21^. Briefly, 1 mL TRIzol Reagent and 200 µL chloroform (08402-55, Nacalai Tesque, Inc.) were added to each sample and then the tubes were vortexed for 15 s and incubated at 25 °C for 3 min. After centrifugation at 12,000*g* for 15 min at 4 °C (Centrifuge 5415R, Eppendorf), the supernatant was transferred to a new tube and 2 µL glycogen (608000, Beckman Coulter) and 500 µL isopropanol (166-04836, Wako Pure Chemical Industries, Ltd.) were added. After incubation at 25 °C for 10 min, the mixture was centrifuged at 12,000*g* for 15 min at 4 °C (Centrifuge 5415R, Eppendorf) and the supernatant was removed. The RNA pellet was washed with 75% ethanol (057-00456, Wako Pure Chemical Industries, Ltd.). The ethanol was removed after centrifugation at 7500*g* for 5 min at 4 °C (Centrifuge 5415R, Eppendorf) to pellet the RNA. The RNA was dried in air for 5 min and then dissolved in 50 µL RNase-free water (AM9937, Life Technologies). The quantity and quality of RNA was assessed using the Agilent 2100 Bioanalyzer 6000 Pico Kit (Agilent Technologies, CA, USA), according to the manufacturer’s protocol. Extracted RNAs were reverse-transcribed into cDNA using the TaqMan MicroRNA Reverse Transcription Kit (4366596, Applied Biosystems, CA, USA). In brief, 6 µL RNA were mixed with 0.4 µL 100 mM dNTP (4367381, Applied Biosystems), 1.6 µL Megaplex RT Primer (4399966, Applied Biosystems), 1.6 µL RT buffer (4319981, Applied Biosystems), 0.2 µL RNase inhibitor (20 U/µL) (4469082, Applied Biosystems), 3 µL MultiScribe reverse transcriptase (50 U/µL) (4308228, Applied Biosystems), 1.8 µL MgCl_2_ (4304898, Applied Biosystems), and 0.4 µL nuclease-free water (AM9937, Life Technologies). The reverse transcription reaction was conducted on a GeneAmp PCR System 2400 thermal cycler (Perkin Elmer, Inc., MA, USA) as follows: 16 °C for 30 min, 42 °C for 30 min, and 85 °C for 5 min. A 2.25 µL sample of RT products was then mixed with 4.5 µL RNase-free water (AM9937, Life Technologies), 7.5 µL QuantStudio 3D Digital PCR Master Mix (A26359, Applied Biosystems), and 0.75 µL TaqMan MicroRNA Assay (186925367, Applied Biosystems). The TaqMan MicroRNA assays used were has-miR21 (Assay ID 000397), has-miR223 (Assay ID: 002295), has-miR155(Assay ID: 002623), and has-miR375 (Assay ID: 000564). The sample mix was loaded onto each chip (A26316, Applied Biosystems) and the chip was placed in a ProFlex System (Thermo Fisher Scientific). The conditions were 96 °C for 10 min, followed by 40 cycles at 56 °C for 2 min, 98 °C for 30 s, and 60 °C for 20 min. After the reaction, the chips were analyzed with a QuantStudio 3D Digital PCR System (Thermo Fisher Scientific) and QuantStudio 3D AnalysisSuite Cloud Software (version 3.1, Thermo Fisher Scientific).

### Statistical analysis

Assays were repeated as at least three independent experiments. The data are presented as the mean ± standard deviation. Significant differences between two groups were calculated with Student *t* tests. Significant differences between more than two groups were calculated with Bonferroni tests^82^. Linear regression analysis was performed to determine simple correlations between two variables. P < 0.05 was considered statistically significant in all statistical analyses.

### Reporting

We have submitted all relevant data of our experiments to the EV-TRACK knowledgebase (EV-TRACK ID: EV190087)^83^.

## Supplementary Information

Supplementary Information.
