# Supplementary material for: Pentapartite fractionation of particles in oral fluids by differential centrifugation
Source: Sci Rep. 2021 Feb 8;11:3326. doi: 10.1038/s41598-021-82451-6 (PMC7870959; doi:10.1038/s41598-021-82451-6)
Supplement: Supplementary file 1 — Supplementary Information. [file 41598_2021_82451_MOESM1_ESM.pdf]

Pentapartite fractionation of particles in oral fluids by differential centrifugation

Chiho Hiraga,[1,2], Satoshi Yamamoto,[3] Sadamitsu Hashimoto,[4] Masataka Kasahara,[3] Tamiko Minamisawa,[1] Sachiko Matsumura,[1] Akira Katakura,[5] Yasutomo Yajima,[6] Takeshi Nomura,[2] Kiyotaka Shiba,[1*]

[1]Division of Protein Engineering, Cancer institute, Japanese Foundation for Cancer Research, Tokyo, Japan; [2]Department of Oral Oncology, Oral and Maxillofacial Surgery, Tokyo Dental College, Chiba, Japan; [3]Department of Pharmacology, Tokyo Dental College, Tokyo, Japan; [4]Laboratory of Biology, Tokyo Dental College, Tokyo, Japan; [5]Department of Oral Pathobiological Science and Surgery, Tokyo Dental College, Tokyo, Japan; [6]Department of Oral Implantology, Tokyo Dental College, Tokyo, Japan (*Correspondence author. Email kshiba@jfcr.or.jp)

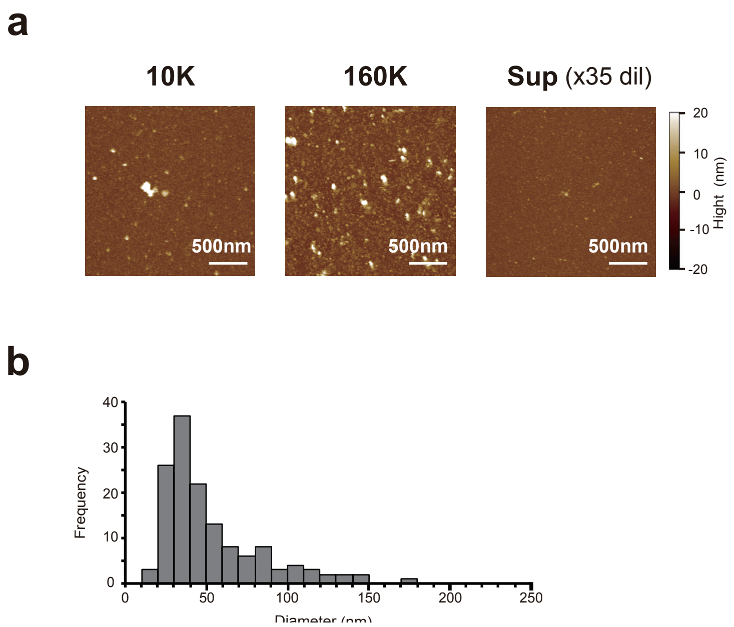

Figure S1. Atomic force microscopic (AFM) observations of each pentapartite fraction from oral fluids (OFs). **a**, AFM images obtained in PBS, as previously described[1]. Briefly, 30 µL of 3-aminopropyltrietrhoxysilane (APTES) (Tokyo Chemical Industry, Tokyo, Japan) was applied to freshly cleaved mica (Ted Pella Inc., CA, USA) under vacuum for 1 h. The samples were diluted with PBS to reach an appropriate concentration. Each 15 µL sample was spread on the APTES-coated mica for 15 h at 4 ºC and the substrates were then washed with PBS (3 times). A 50 µL volume of PBS was added at the time of measurement and the sample was imaged with atomic force microscopy (MFP-3D-SA-J, Asylum Research, an Oxford Instruments, CA, USA) under aqueous conditions in tapping mode with silicon tips (BL-AC40TS-C2, Olympus Corporation, Tokyo, Japan). Scale bar: 500 nm. **b**, Histogram of the diameter of a particle in the 160K fraction estimated by AFM: mean = 52.7 ± 30.7 nm, mode = 40.6 nm, max = 175.1 nm and min = 12.4 nm. Topographic height and phase images were recorded at 2 × 2 µm2 and 512 × 256 pixels at a scan rate of 0.30 Hz. A flattening process was applied and image processing was performed using image analysis software (SPIP 6.3.6) (Image Metrology, Kongens Lyngby, Denmark). The detection thresholds were > 2 nm for height and > 7.5 nm for Zmax. The histogram was made from data generated for more than 100 round shape objects.

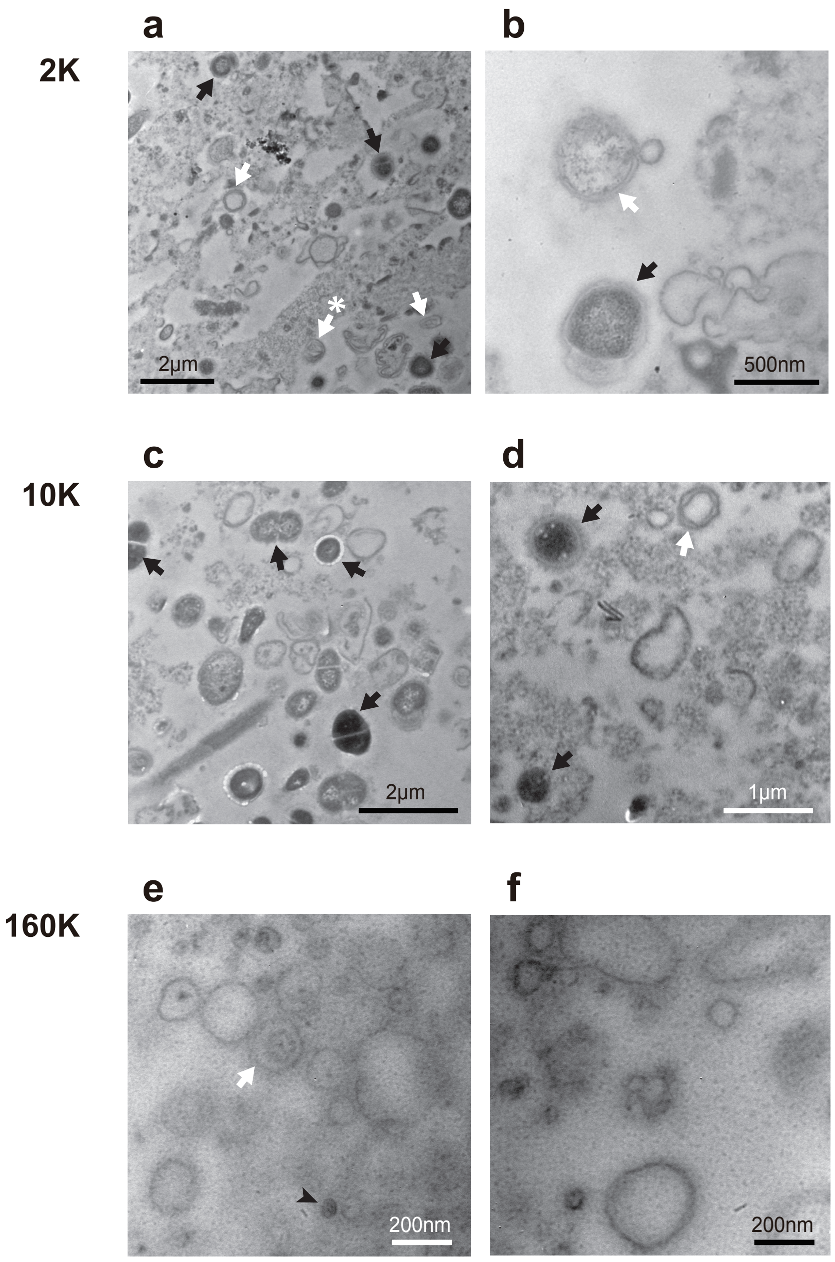

Figure S2. Transmission electron microscopy (TEM) for 2K (**a**, **b**), 10K (**c** , **d**), and 160K (**e** , **f**) fractions from oral fluids (OFs). Ultra-thin sections of the pellets obtained after centrifugation were stained with uranyl acetate and lead citrate (see Materials and methods in the main text). Vesicles with multiple bilayers are labeled with white arrows, some of which contained intraluminal vesicle structures (labeled with asterisks). The high-density particles may represent oral bacterial cells (black arrows) and their EVs (black arrowheads).

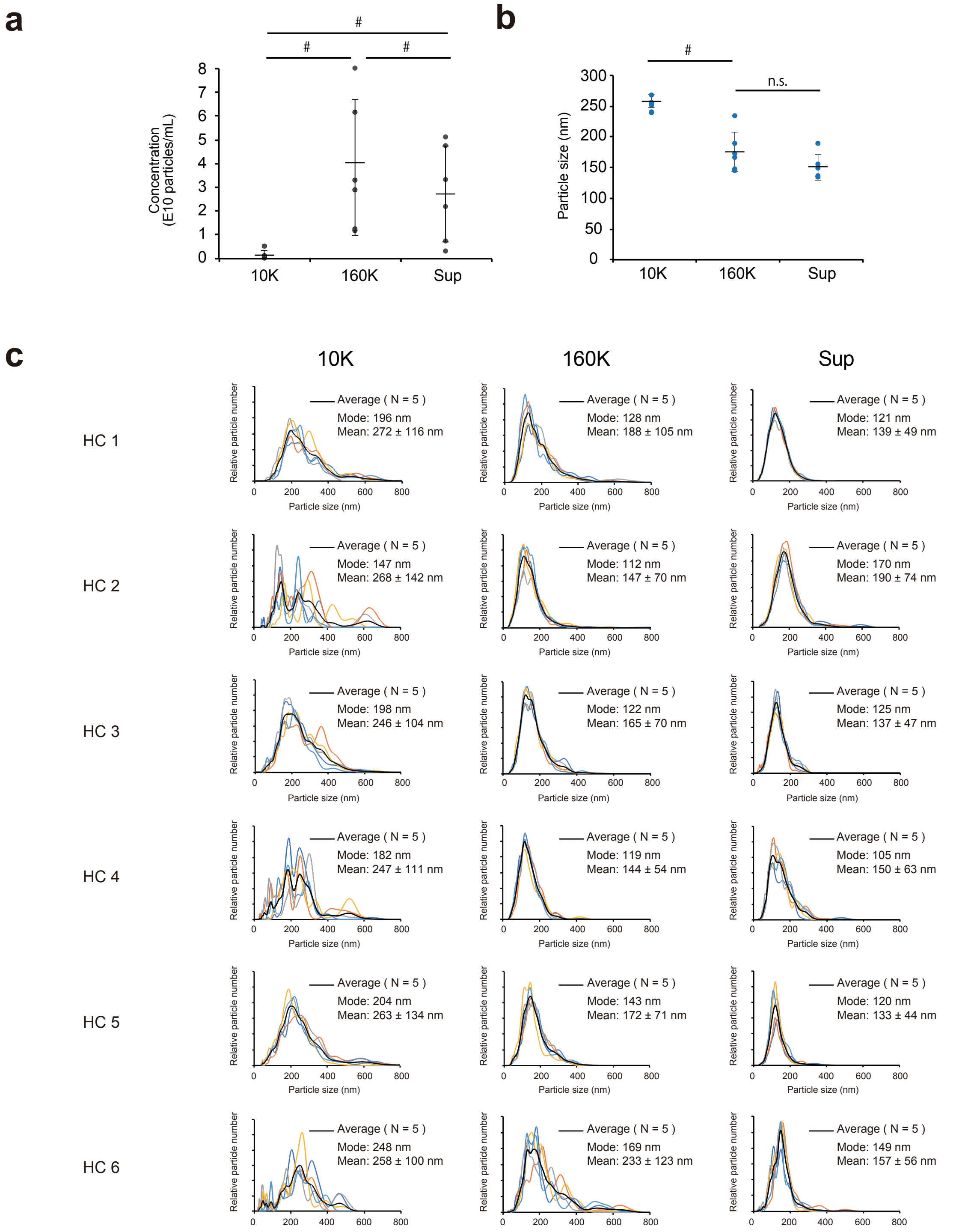

Figure S3. The distributions of the numbers and sizes of EVs contained in each fraction determined by a nanoparticle tracking analysis (NTA). **a**, Concentration of particles contained in each fraction. **b**, Mode sizes of particles. Dots indicate the mean value of the five measurements for each subject and the mean value obtained from six subjects are represented as solid horizontal lines. The Bonferroni test was used to evaluate statistical significance. $p < 0.05$ was considered as statistically significant. n.s.; not significance. **c**, Distributions of sizes of particles for each subject. Five measurements and their average histograms are shown by thin lines and thick black lines, respectively.

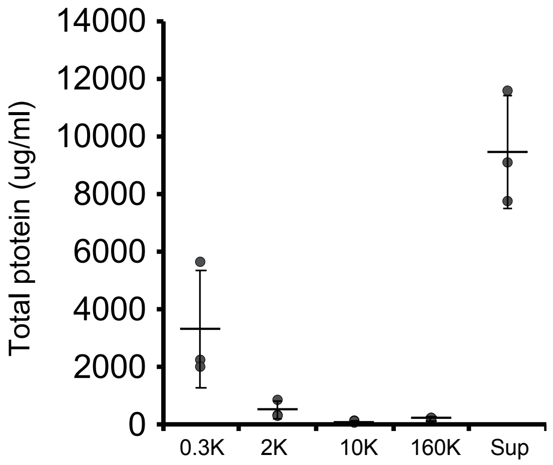

Figure S4. Protein concentrations assessed by a bicinchoninic acid (BCA) assay kit (23228, 1859078, Thermo Fisher Scientific, USA), using bovine serum albumin (23209, Thermo Fisher Scientific) as a standard. Dots indicate the mean value of the three measurements individually in three healthy volunteers. The mean value of groups is represented as solid horizontal lines.

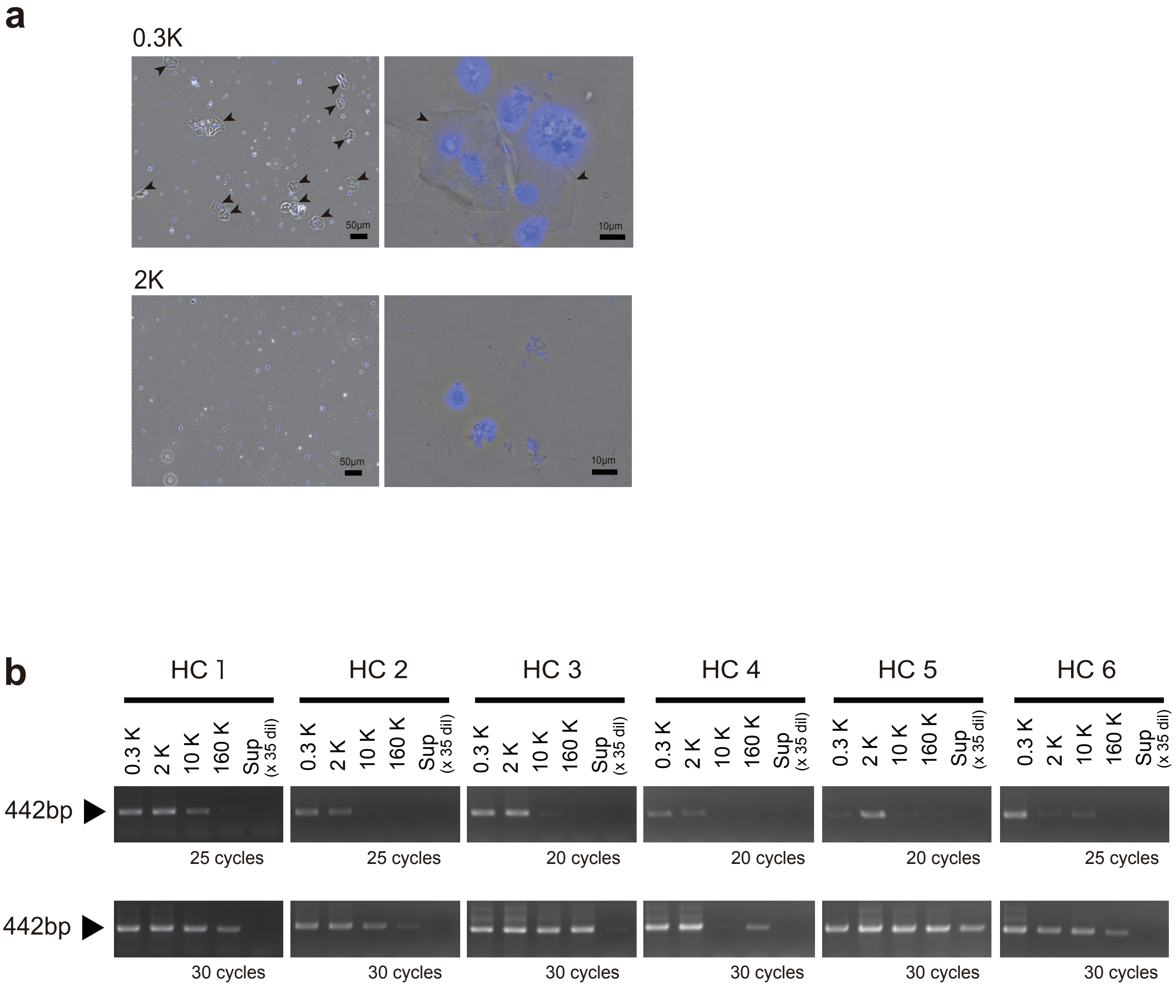

Figure S5. a, Representative images of fractions stained with 4',6-diamidino-2-phenylindole (DAPI) in phase-contrast micrographs. Arrowheads indicate the oral squamous cells. **b**, Semi-quantitative estimation of the presence of bacterial DNA in each fraction by targeting PCR for 16S ribosomal DNA[2]. In brief, oligonucleotide primers (Forward;5'-TCCTACGGGAGGCAGCAGT-3' and Reverse;5'-GGACTACCAGGGTATCTAATCCTGTT-3') were used for PCR carried out in 25 μL reaction volumes containing 10 μM primers, 2.5 mM dNTP (TaKaRa), Taq DNA polymerase (50 U/μL) (11418432001, Roche, Basel, Switzerland), 10 × PCR Reaction buffer (10356000, Roche) and nuclease-free water (AM9937, Life Technologies) with the following conditions: 94 °C for 2 min, followed by 30 cycles of 94 °C for 30 sec, 59 °C for 1 min, and 72 °C for 1 min, and 72 °C for 7 min. The amount of bacterial DNA derived in each fraction was inspected visually following electrophoresis on 1% agarose gels and Lumi Vision LPR-130 (TAITEC, Saitama, Japan). With increased cycles of PCR, the 10K and 160K fractions also gave the PCR products, suggesting that small amounts of bacterial DNA were recovered in these fractions.

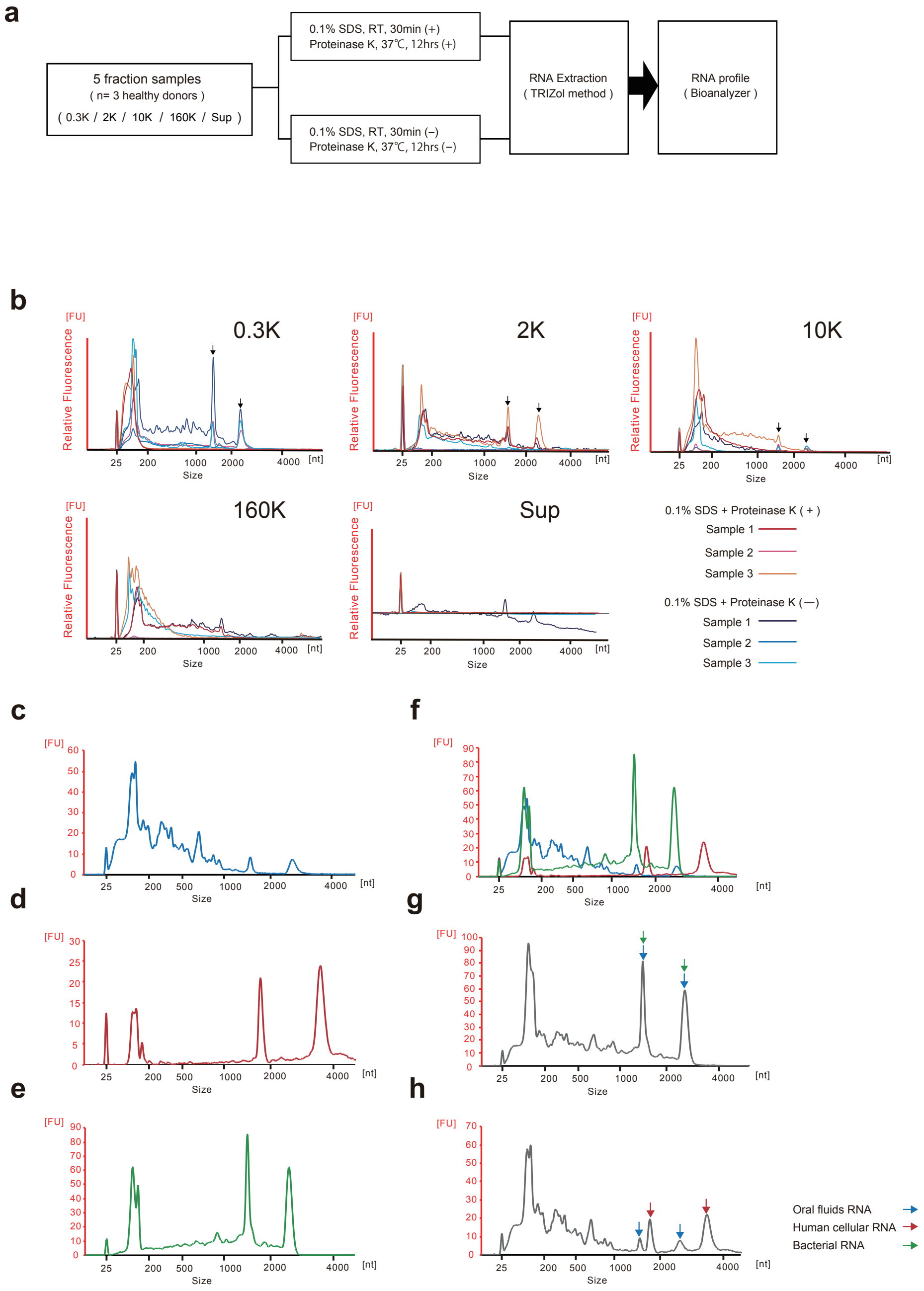

Figure S6. Optimization of methods to extract RNA. **a**, Work flow of RNA extraction steps. Each fraction obtained from differential centrifugation was treated with and without 0.1% SDS for 30 min at 25ºC and 100 µg/mL Proteinase K Solution (Wako Pure Chemical Industries, Ltd) for 12 h at 37ºC. The total RNA was then extracted using TRIzol Reagent (15596-018, Life Technologies), as previously described[3]. Briefly, 1 mL TRIzol reagent and 200 µL chloroform (08402-55, Nacalai Tesque, Inc.) were added to the sample and the mixture was vortexed for 15 sec and incubated at 25ºC for 3 min. After centrifugation at 12,000 g for 15 min at 4ºC (Centrifuge 5415R, Eppendorf), the supernatant was transferred to a new tube and glycogen (608000, Beckman Coulter) and 500 µL isopropanol (166-04836, Wako Pure Chemical Industries, Ltd.) were added. After incubation at 25ºC for 10 min, the mixture was centrifuged at 12,000 g for 15 min at 4ºC (Centrifuge 5415R, Eppendorf) and the supernatant was removed. The RNA pellet was washed with 75% ethanol (057-00456, Wako Pure Chemical Industries, Ltd.). After centrifugation at 7,500 g for 5 min at 4ºC (Centrifuge 5415R, Eppendorf) and removal of the ethanol, RNA was dried in air for 5 min and then dissolved in 50 µL RNase-free water (AM9937, Life Technologies). The quantity and quality of RNA was assessed using Agilent 2100 Bioanalyzer 6000 Pico Kit (Agilent technologies, CA, USA) according to the manufacturer's protocol. Control cellular RNAs were prepared from the MCF-7 (human breast cancer) cell line using TRIzol Reagent (Life Technologies), according to the manufacture's protocol. Control bacterial RNAs were prepared from Escherichia coli DH5α (DNA-903, Toyobo Life Science, Osaka, Japan) by the TRIzol method. Isolated RNA was profiled with an Agilent 2100 Bioanalyzer (Agilent Technologies). **b**, The distributions of total RNA with and without proteinase K and 0.1% SDS were analyzed using the RNA 6000 Pico Kit and the Agilent 2100 Bioanalyzer. Black arrows indicate ribosomal RNAs. The origin of these ribosomal RNAs was analyzed using the RNA isolated from the 0.3K fraction of OFs **c**, from human cell line (MCF-7) **d**, from bacteria (DH5α) **e**, the mixture of OFs, MCF-7 and (DH5α) **f**, the mixture of OFs and MCF-7 **g**, and the mixture of OFs and MCF-7 (**h**). In the figure, blue, red, and green arrows indicate OFs, the human cell line, and bacterial rRNAs, respectively. The separated peak in (**h**) indicates that the rRNA in OFs was of bacterial origin.

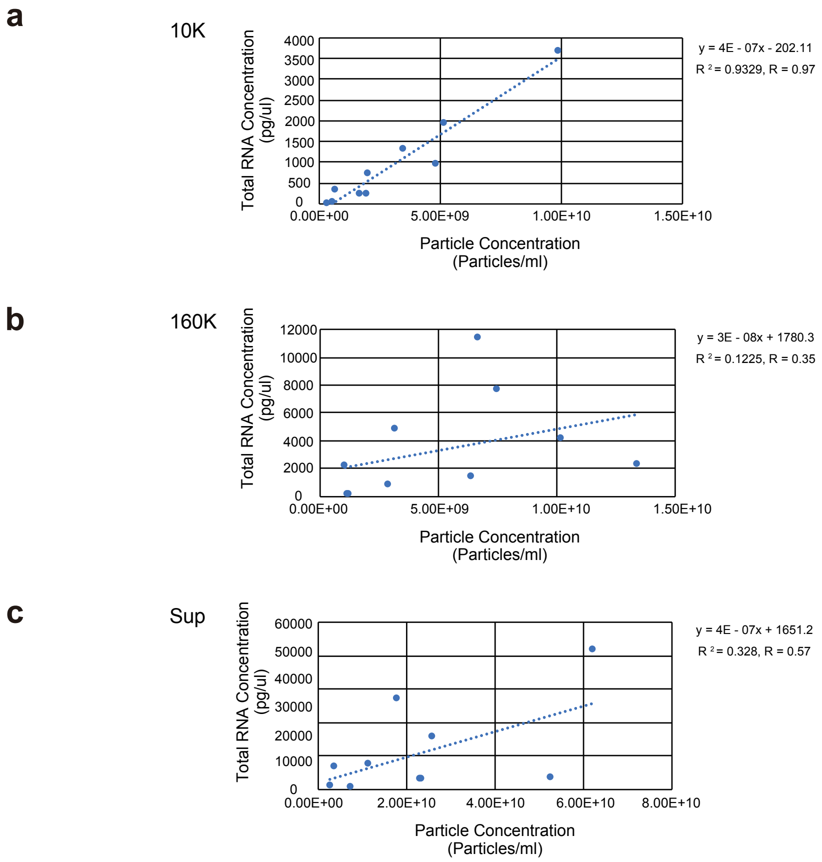

Figure S7. Validation of total RNA extraction from the pentapartite fractions from oral fluids (OFs). **a**, Linear regression analysis between total RNA concentration and particle concentration (n = 10). Correlation between total RNA concentration and particle numbers in the 10K fraction (R = 0.97; P = 5.7 x 10^{-6}), **b**, the 160K fraction (R = 0.35; P = 0.3215) and **c**, supernatant (R = 0.57; P = 0.0835). Linear regression analysis was performed to determine whether a simple correlation existed between two variables. $p < 0.05$ was considered as statistically significant.

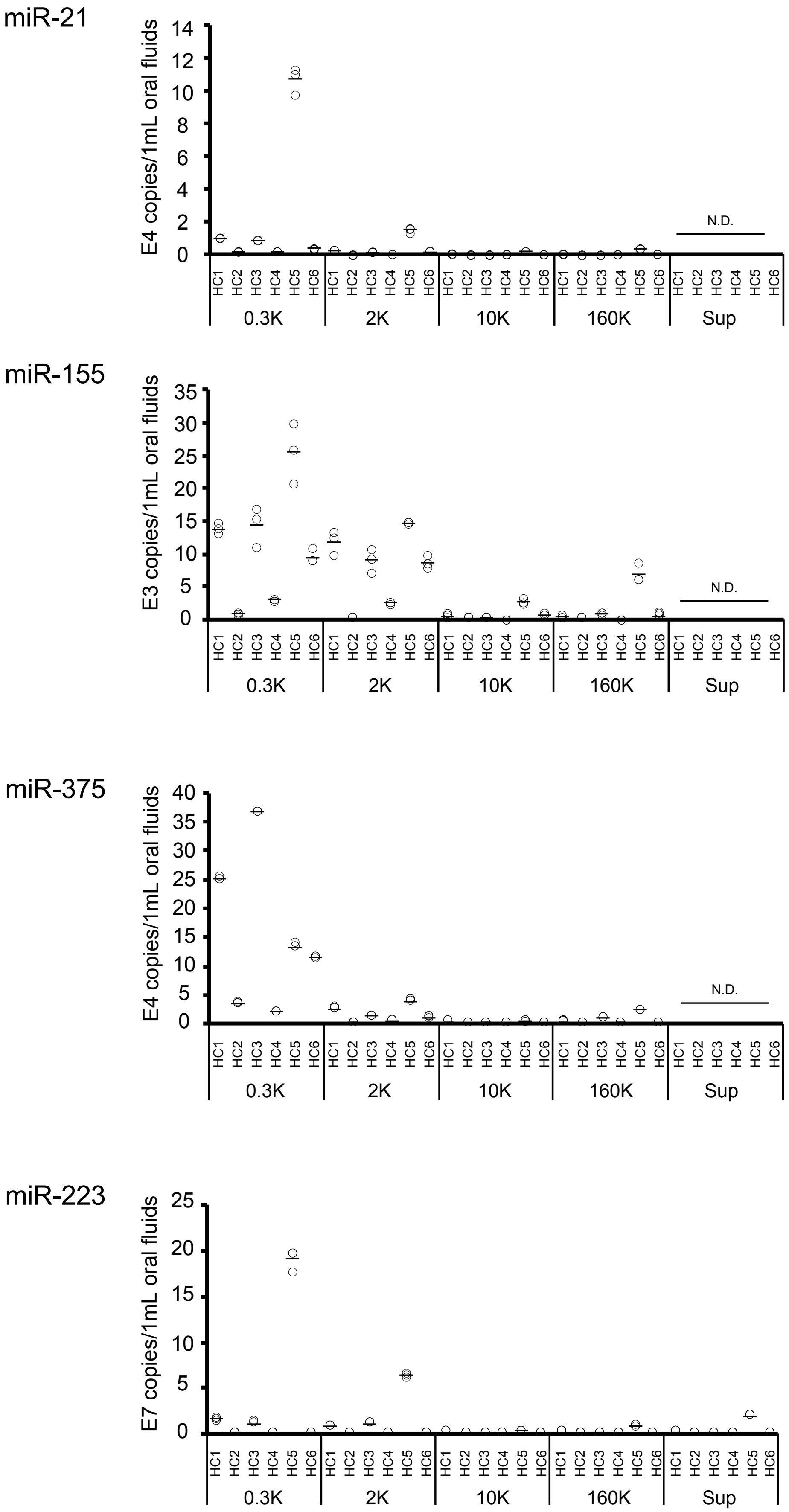

Figure S8. The expression levels of 4 microRNAs (miR-223, 21, 115, and 375) in 6 healthy donors. The copy numbers of microRNAs per mL of oral fluid (OF) derived from healthy controls (n=6 donors) were determined using chip-based Quant Studio 3D digital PCR. The experiments were done in triplicate. Dots indicate the values of the three measurements. The mean values of three measurements are represented as solid horizontal lines.

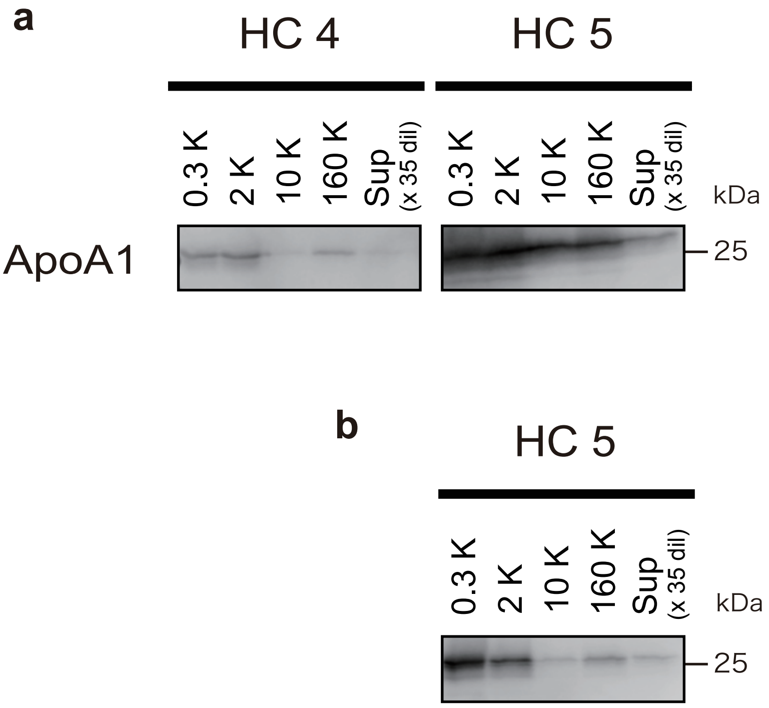

Figure S9. The expression levels of the HDL protein marker ApoA1. **a**, Western blot analyses of 5 fractions derived from healthy controls (HC4 and HC5). Equal sample volumes (18μL) were loaded for HC4 and HC5. **b**, HC5 sample volumes (2μL) were loaded in each lane. Western blot analyses were performed as described in the Materials and Methods in the main text, using goat anti-ApoA1(11A-G2B, Cosmobio Tokyo, Japan; 1:1000 dilution). Each fraction is shown at the top. The numbers on the right indicate $\times 10^{-3}$ of MW marker.

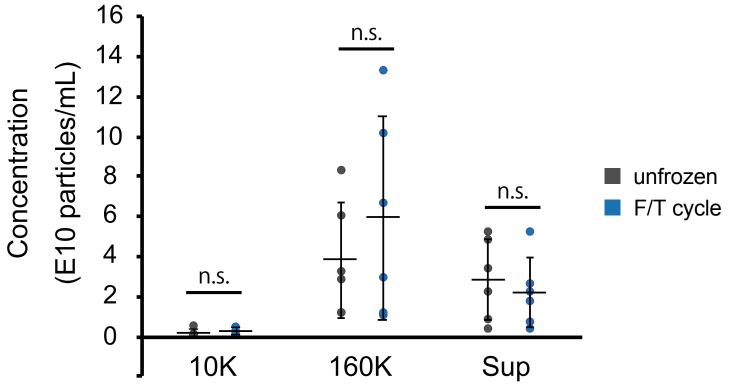

Figure S10. Comparison of particles before (unfrozen) and after one freeze/thaw (F/T) cycle. The particle concentrations were determined using nanoparticle tracking analysis (NTA) in each fraction purified from fresh oral fluids and cryopreservation oral fluids (n = 6 in each group).

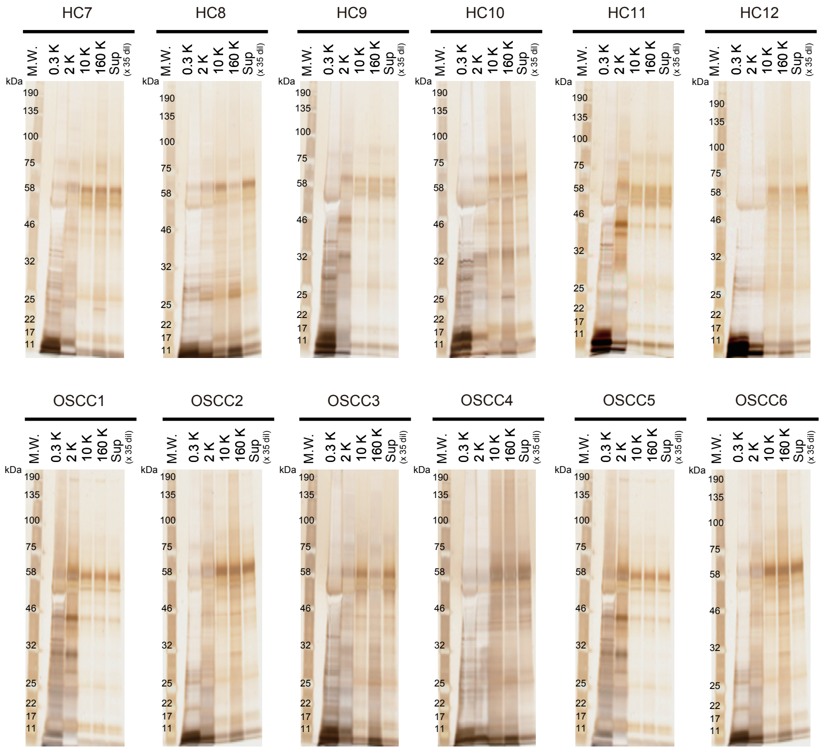

Figure S11. Silver staining analysis of EVs from 6 patients with OSCC and 6 healthy donors. Gels were fixed in acetic acid (00212-85, Nacalai Tesque, Inc.) and silver stained using Sil Best Stain One (06865-81, Nacalai Tesque, Inc.) according to manufacturer's instructions.

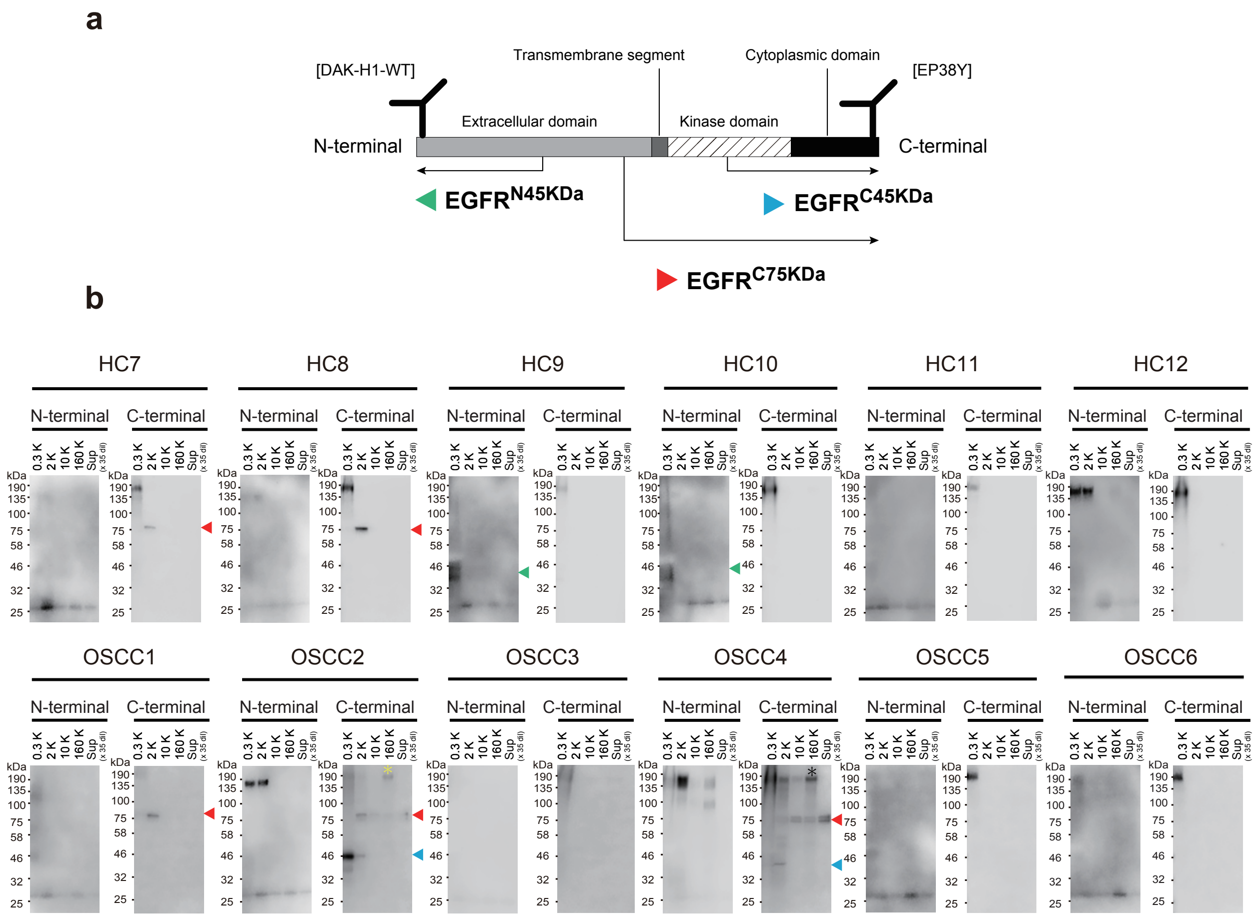

Figure S12. The expression levels of EGFR. **a**, Schematic domain structure of EGFR and maps of three shorter derivatives, EGFRC^{75KDa}, EGFRC^{45KDa} and EGFRN^{45KDa}. Antibodies used for N-terminal and C-terminal detection were DAK-H1-WT and EP38Y, respectively. **b**, Western blot analyses of pentapartite fractions from oral fluids (OFs) from healthy controls (HC 1 to 6) and patients with OSCC (OSCC1 to 6). The signals corresponding to EGFRC75KDa, EGFRC45KDa and EGFRN45KDa are shown by red, cyan, and green arrowheads, respectively. Each fraction is shown at the top. Numbers on the left indicate the molecular weight ×10^{-3} of MW markers. Note the data for the C-terminal antibody were identical to the ones shown in Figure 3.

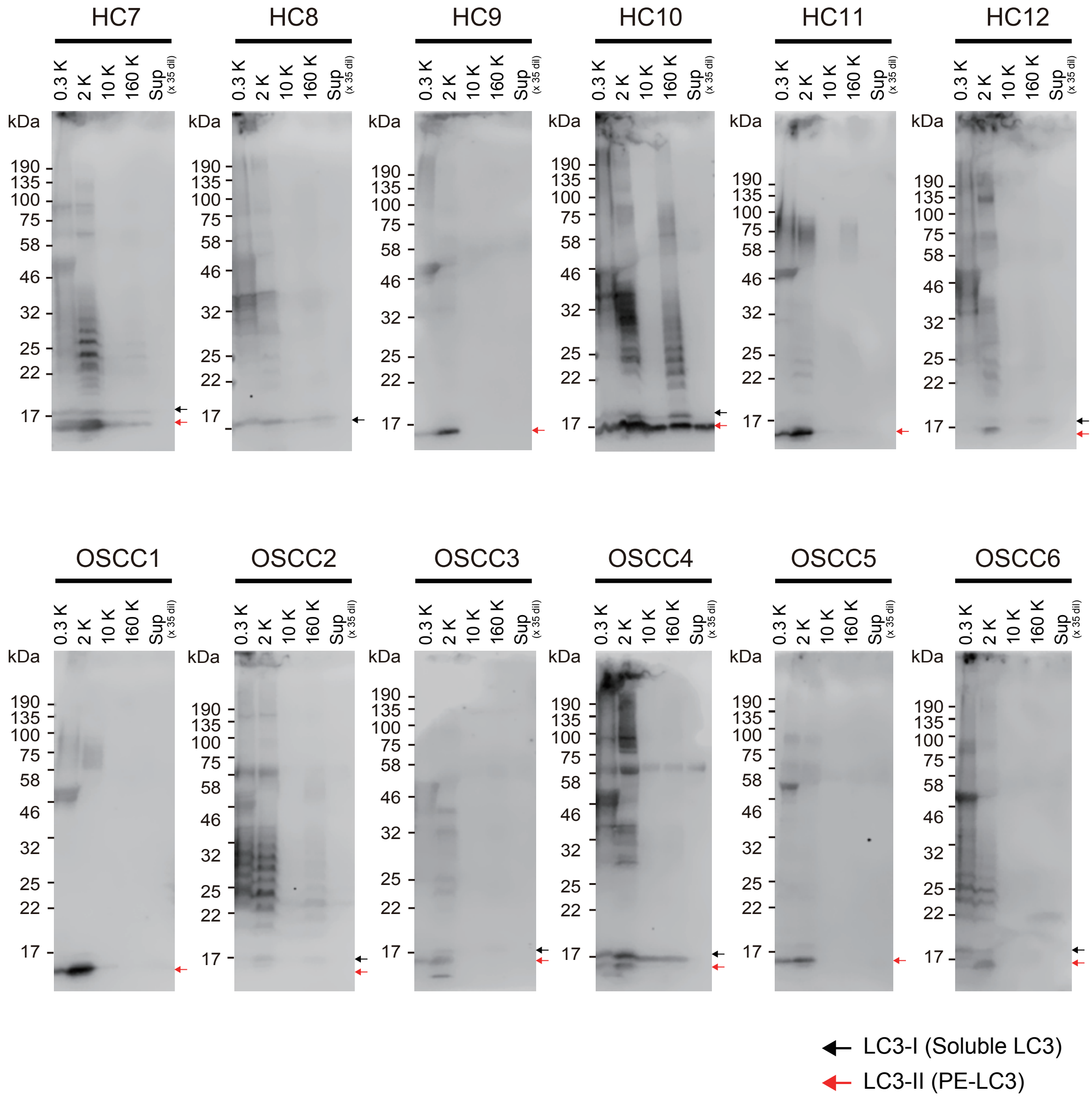

Figure S13. The expression levels of the autophagosome marker LC3. Each fraction is shown in atf top. Black arrows and red arrows indicated LC3-I and LC3-II, respectively. Numbers on the right indicate molecular weight ×10^{-3} of MW markers. For detection of LC3, proteins were separated by 12.5 % SDS-polyacrylamide gel electrophoresis (Extra PAGE One Precast Gel, Nacalai Tesque) in SDS running buffer and electro-transferred onto 10 cm × 10 cm PVDF membranes (IB401002, Invitrogen). The membrane was blocked in 10 mL of 1 % skim milk (Difco™ skim milk, 232100, Becton, Dickinson and Company, NJ, USA) in TBS-T and incubated for 18 h at 4 °C to prevent nonspecific binding. Anti LC3 antibody and secondary antibody were diluted in 1% skim milk in TBS-T. The same was then used with the other antibodies.

References

1. Matsumura, S. et al. Subtypes of tumour cell-derived small extracellular vesicles having differently externalized phosphatidylserine. J Extracell Vesicles 8, 1579541, doi:10.1080/20013078.2019.1579541 (2019).
2. Kuehn, M. J. & Kesty, N. C. Bacterial outer membrane vesicles and the host-pathogen interaction. Genes Dev 19, 2645-2655, doi:10.1101/gad.1299905 (2005).
3. Iwai, K., Minamisawa, T., Suga, K., Yajima, Y. & Shiba, K. Isolation of human salivary extracellular vesicles by iodixanol density gradient ultracentrifugation and their characterizations. J Extracell Vesicles 5, 30829, doi:10.3402/jev.v5.30829 (2016).

Table S1. Clinicopathological features of healthy volunteers (HC) and patients with OSCC.

Sample	Age	[a]Sex	Location	[b]Smoke	[c]Alcohol	[d]pTNM	[d]Stage	[e]Metastasis
HC1	31	F	-	No	Yes	-	-	-
HC2	37	F	-	No	No	-	-	-
HC3	44	F	-	No	No	-	-	-
HC4	30	M	-	Yes	No	-	-	-
HC5	53	F	-	No	No	-	-	-
HC6	31	M	-	No	Yes	-	-	-
HC7	62	M	-	No	No	-	-	-
HC8	53	F	-	No	No	-	-	-
HC9	49	M	-	No	Yes	-	-	-
HC10	69	M	-	Yes	Yes	-	-	-
HC11	49	M	-	Yes	Yes	-	-	-
HC12	61	M	-	No	No	-	-	-
OSCC1	69	F	Tongue	No	No	pT4aN0 M0	IV	No
OSCC2	55	F	Gum	No	No	pT4aN2cM0	IV	No
OSCC3	67	M	Gum	No	Yes	pT4aN0 M0	IV	Yes
OSCC4	60	M	Tongue	No	Yes	pT4aN2bM0	IV	No
OSCC5	67	F	Gum	Yes	Yes	pT3N0M0	III	No
OSCC6	24	F	Tongue	No	No	pT3N0M0	III	No

[a]F; female, M; male

[b]Those who had ever smoked cigarettes (including smokeless cigarette).

[c]Those who drink at least once per week.

[d]Based on the 8th Edition of the Union for International Cancer Control (UICC).

[e]Those who had secondary cervical lymph node metastasis.

Original data for western blots

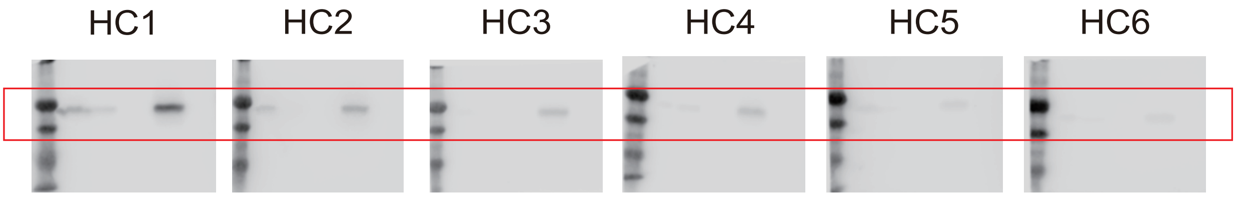

For Fig. 2**a** CD9 (The same gels gave two blots for CD9 and CD81 in Fig. 2)

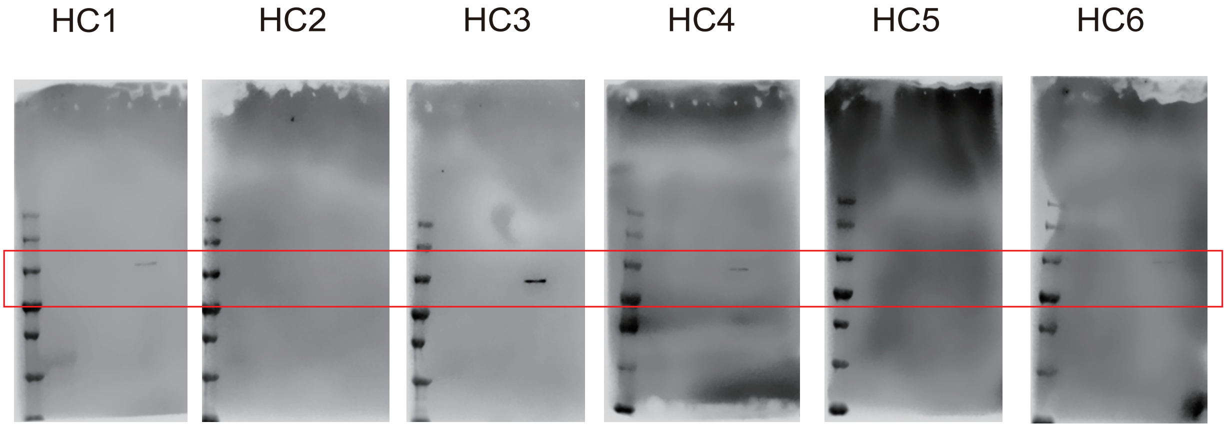

For Fig. 2**a** Alix (The same gels gave blots for Alix and AQP5 in Fig. 2)

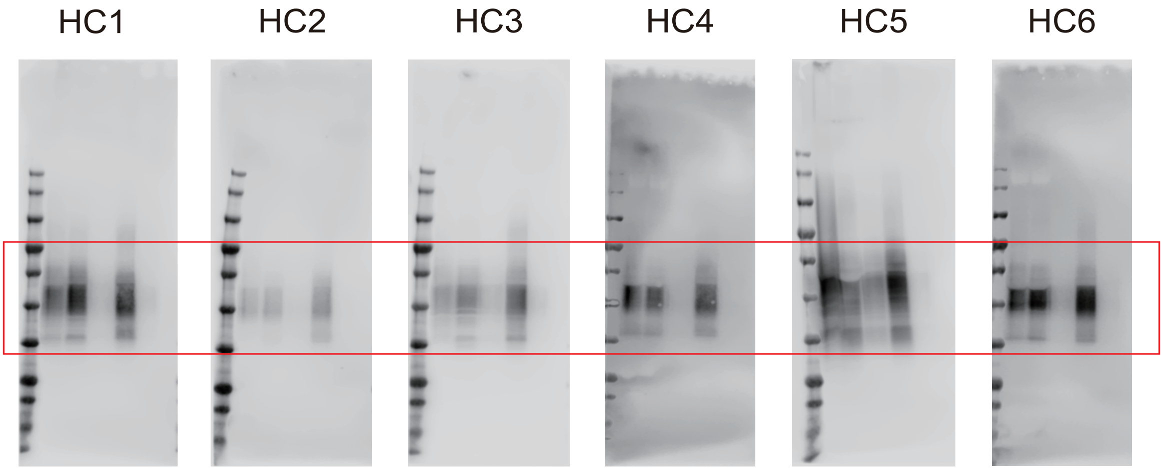

For Fig. 2**a** CD63

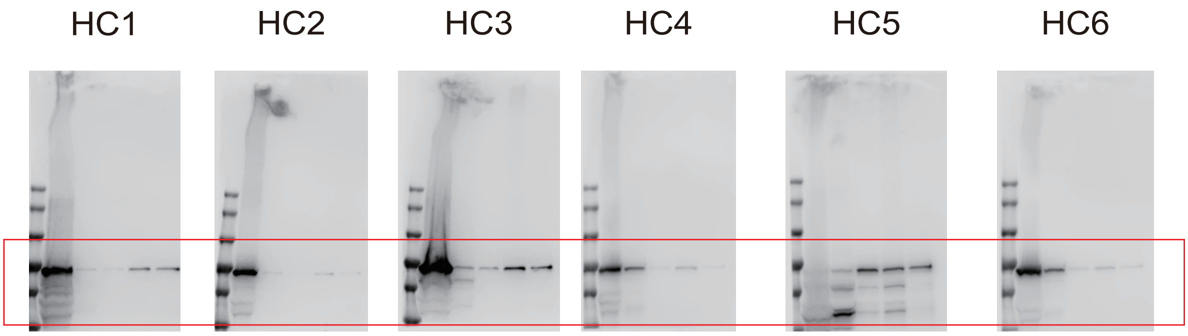

For Fig. 2**a** HSP70

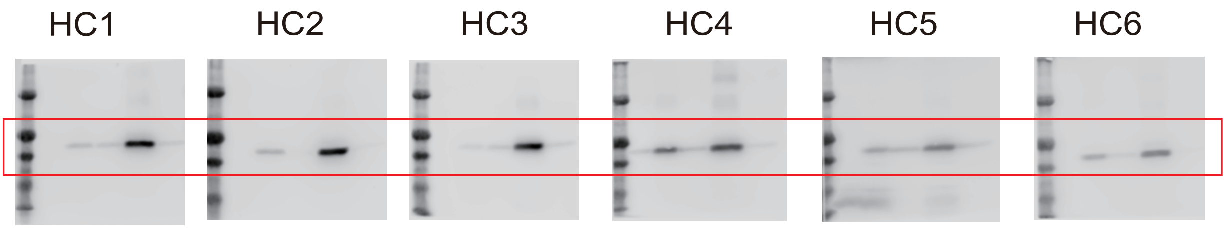

For Fig. 2**a** AQP5 (The same gels gave blots for Alix and AQP5 in Fig. 2)

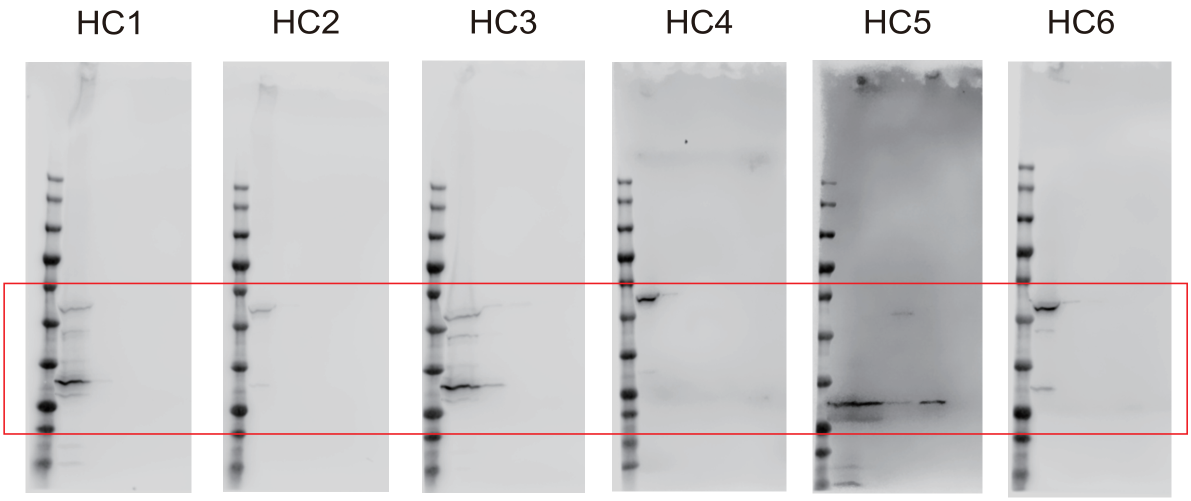

For Fig. 2**a** ATP5A

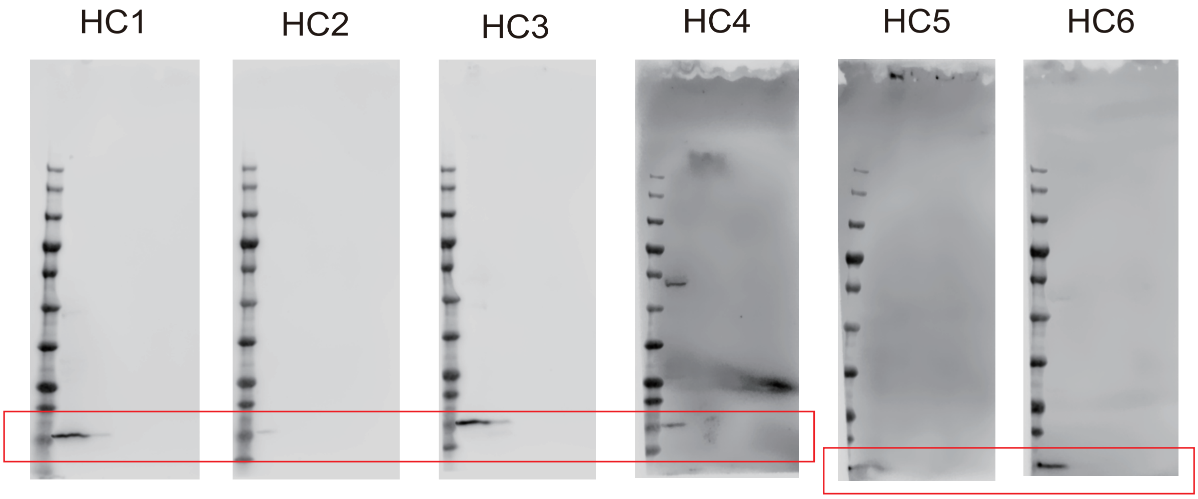

For Fig. 2**a** Histone H2B

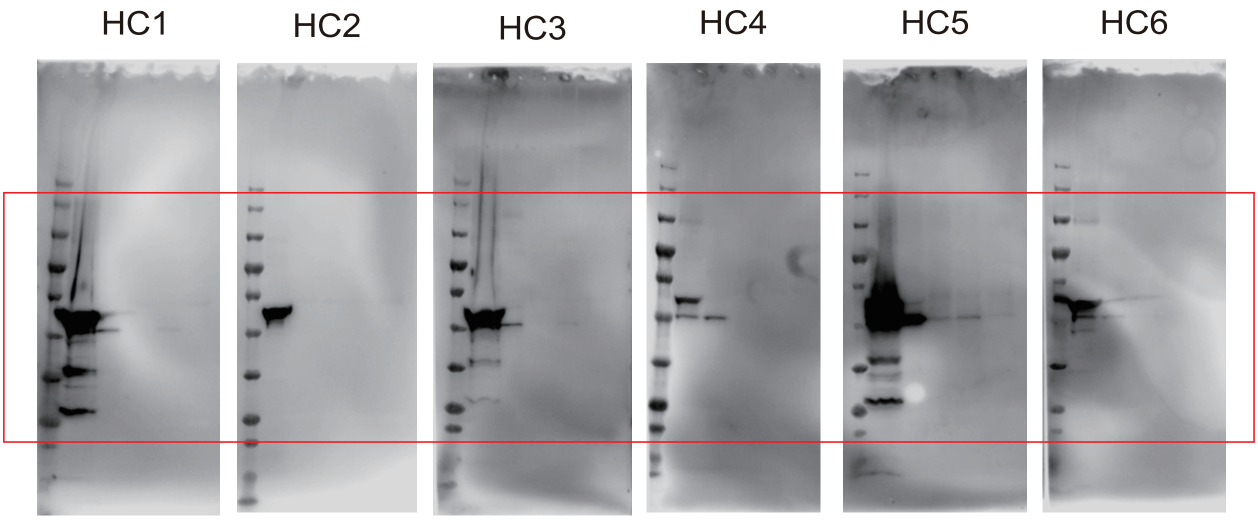

For Fig. 2**a** Ago2

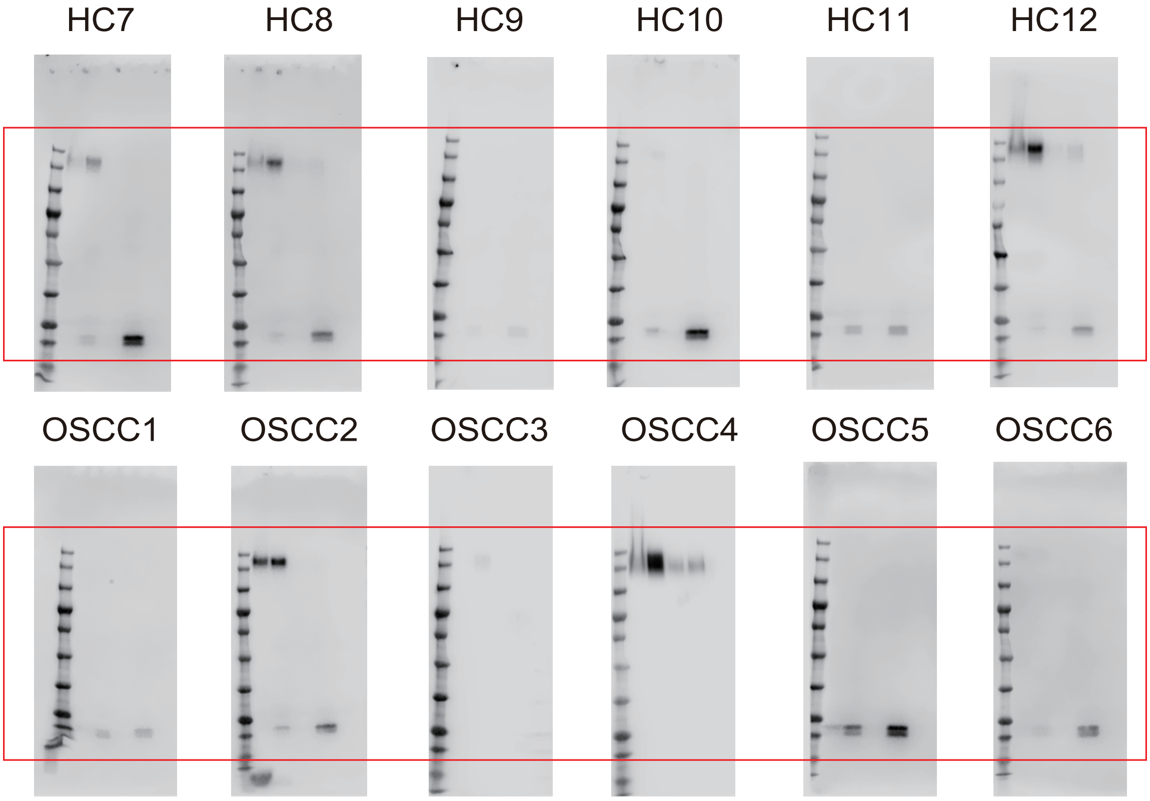

For Fig. 4**a** CD81

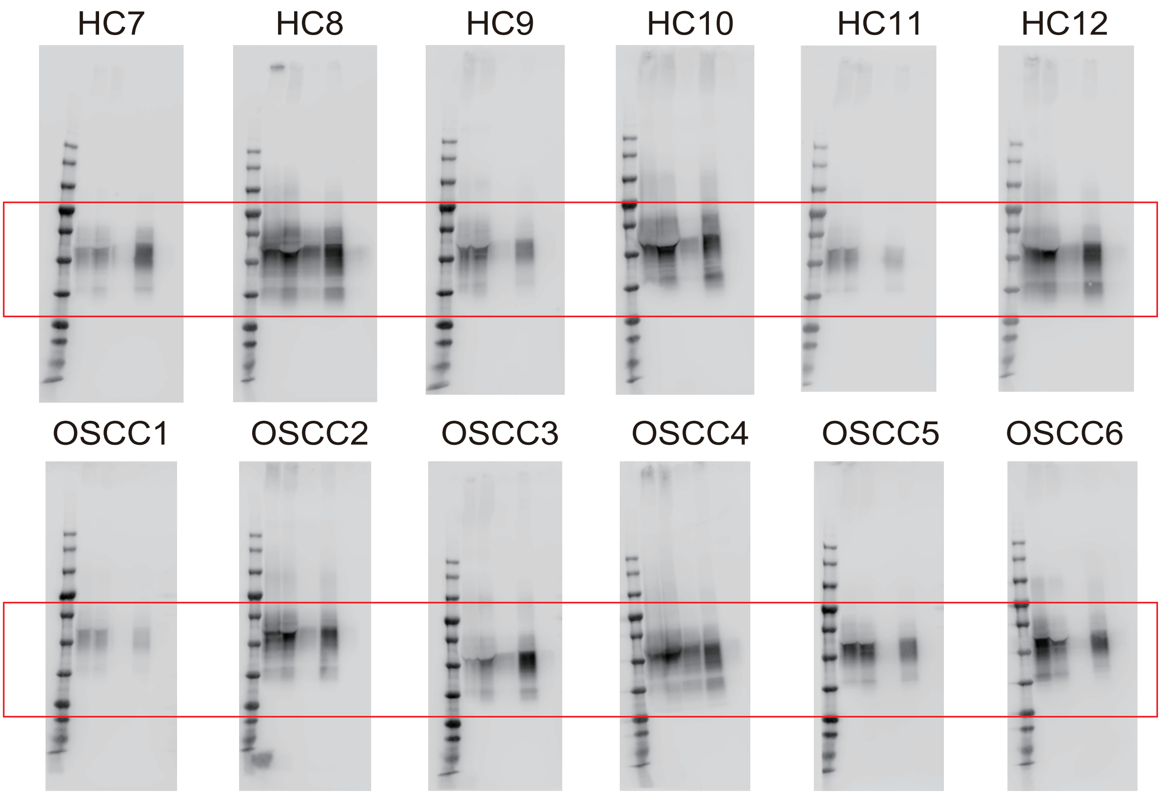

For Fig. 4**a** CD63

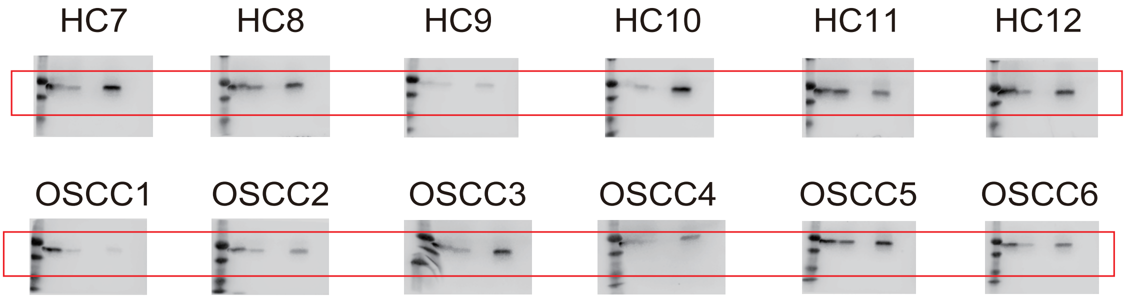

For Fig. 4**a** CD9 (The same gels gave blots for CD9 and Alix in Fig. 4)

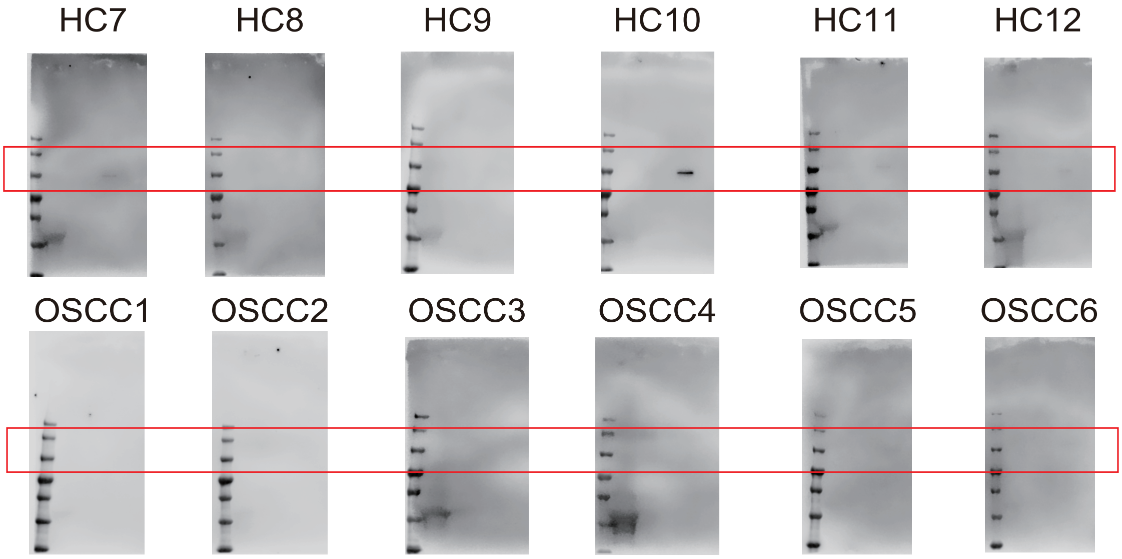

For Fig. 4**a** Alix (The same gels gave blots for CD9 and Alix in Fig. 4)

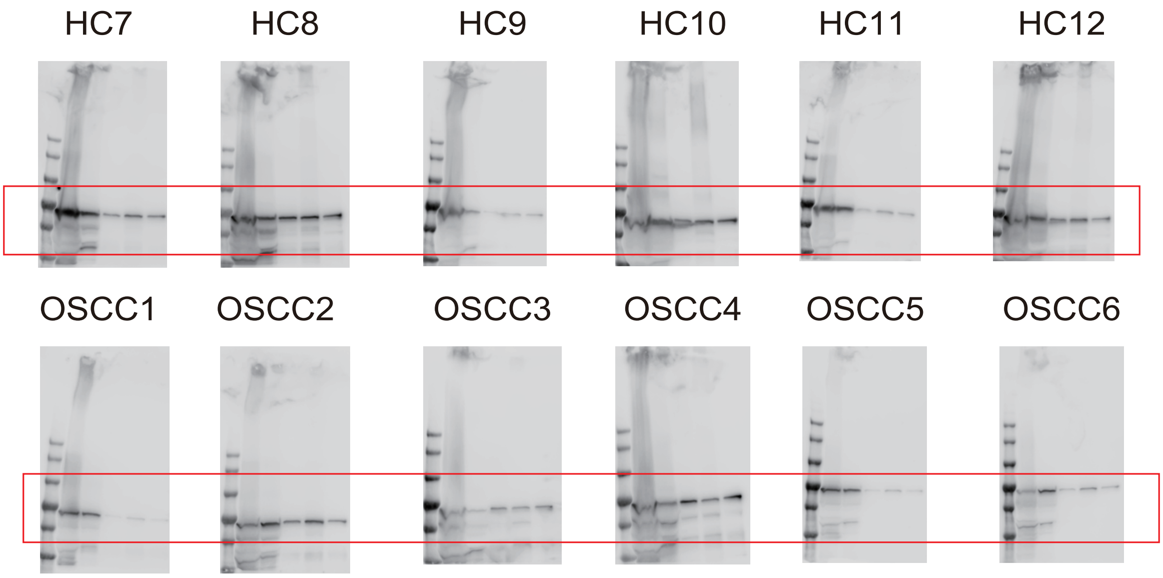

For Fig. 4**a** HSP70 (The same gels gave blots for HSP70 and AQP5 in Fig. 4)

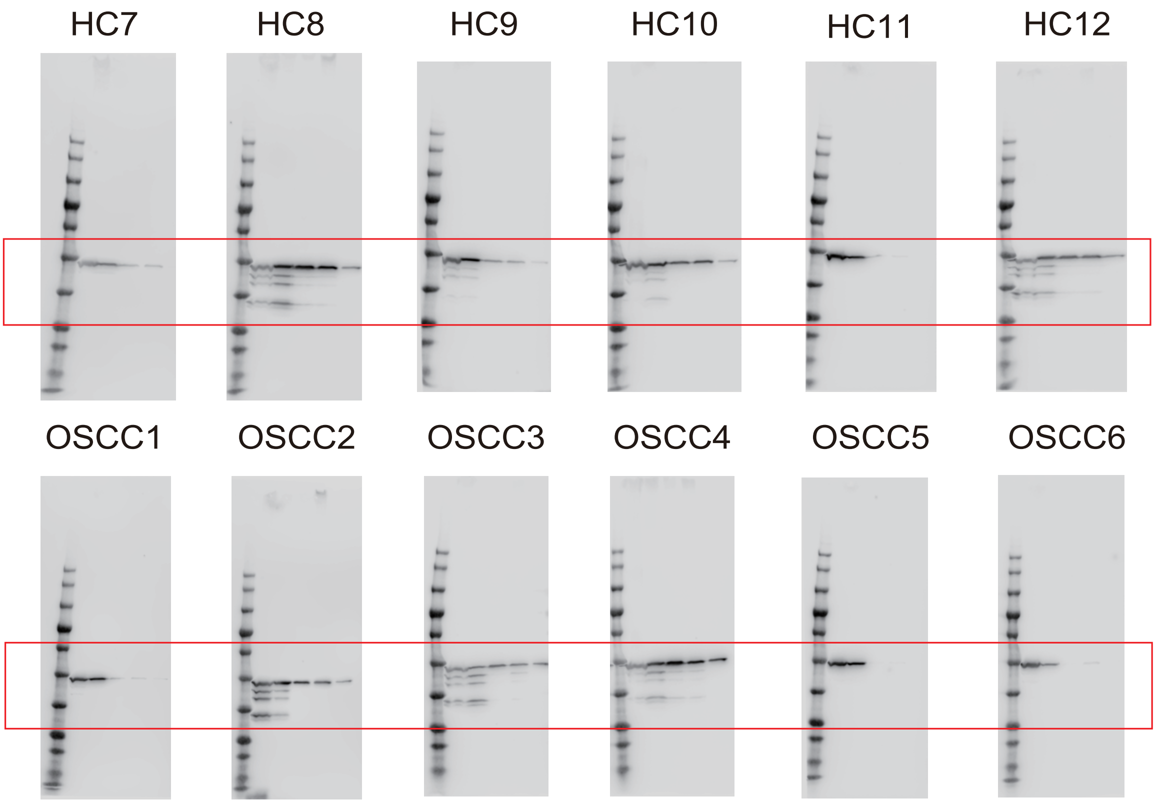

For Fig. 4**a** beta actin

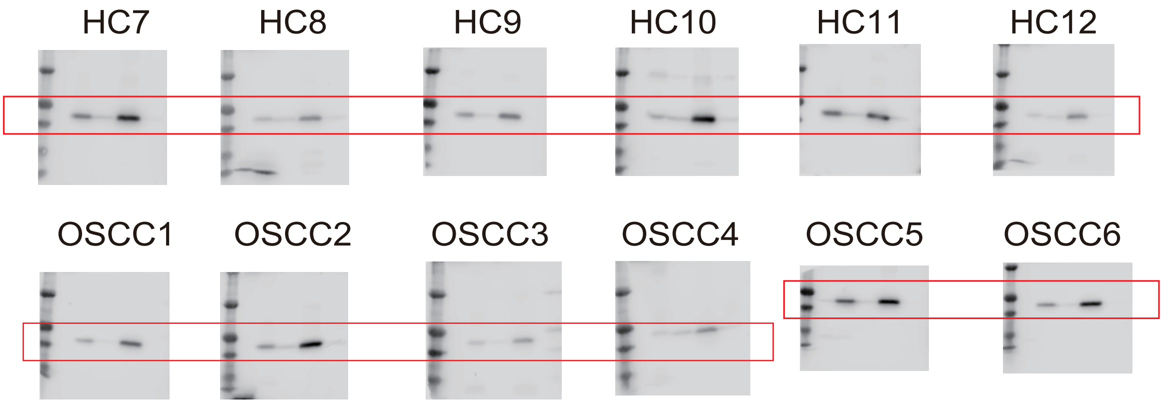

For Fig. 4**a** AQP5 (The same gels gave blots for HSP70 and AQP5 in Fig. 4)

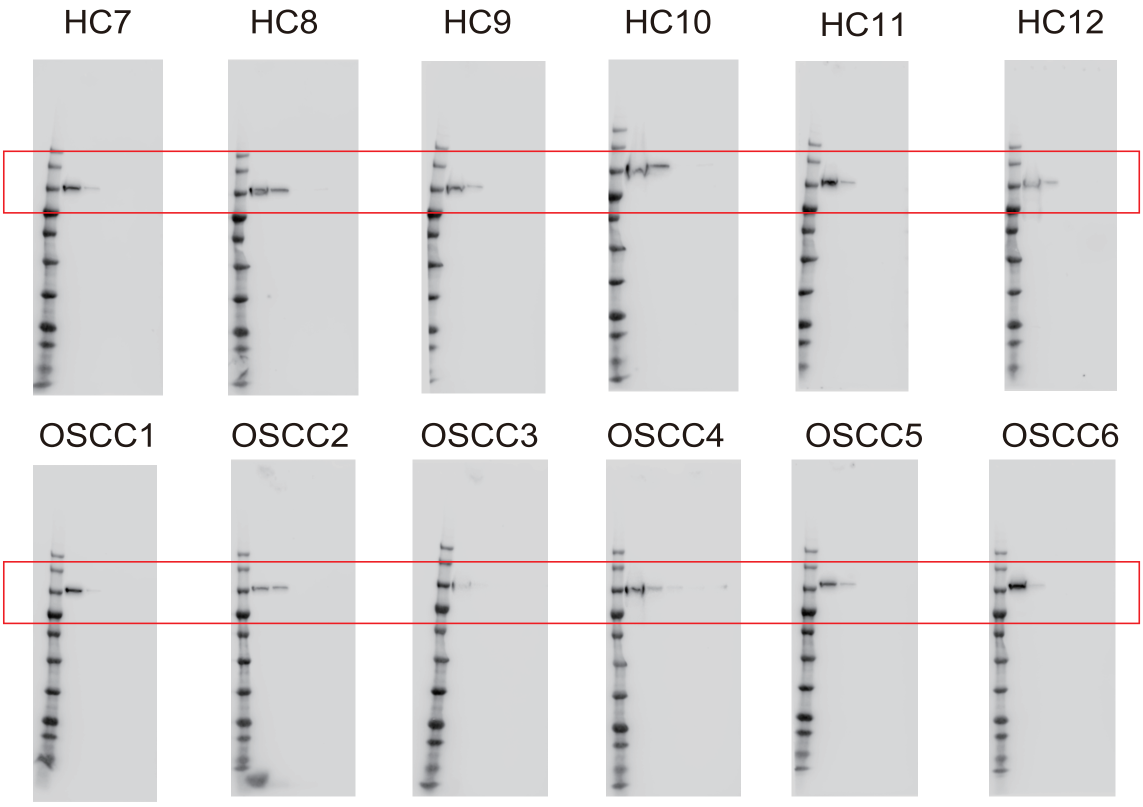

For Fig. 4**a** ACTN4

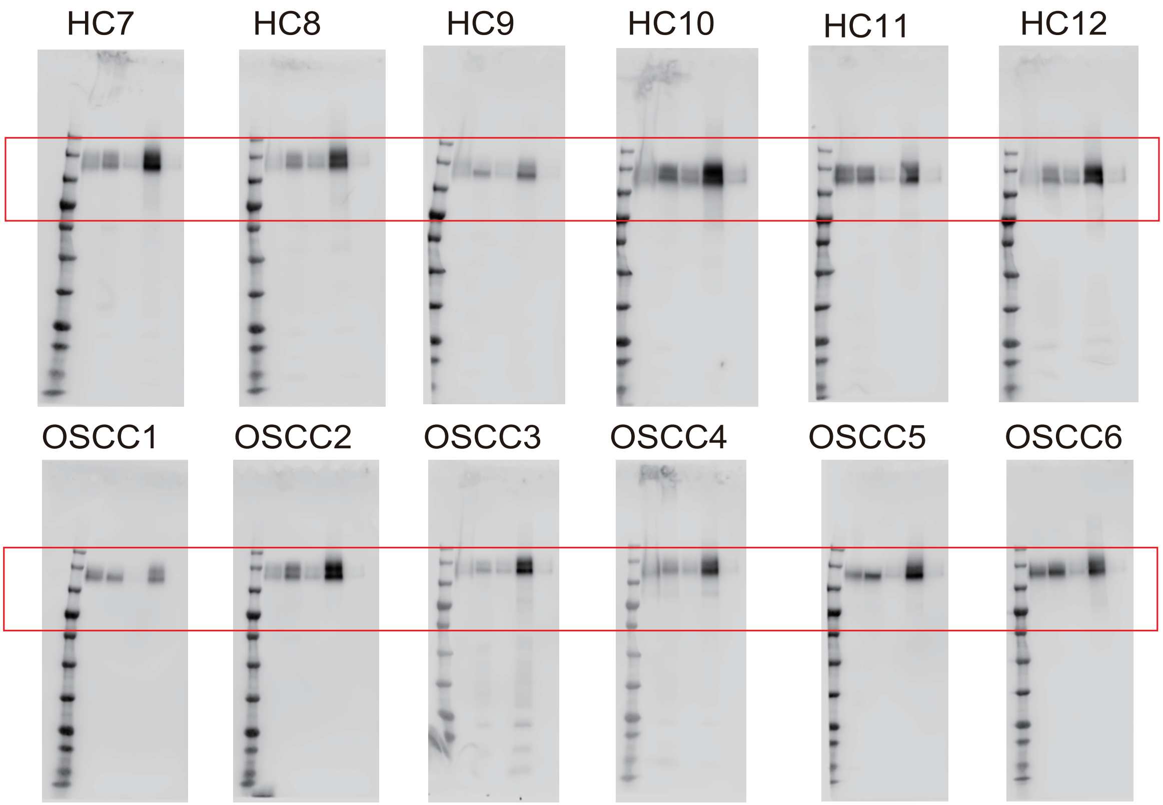

For Fig. 4**a** CD133

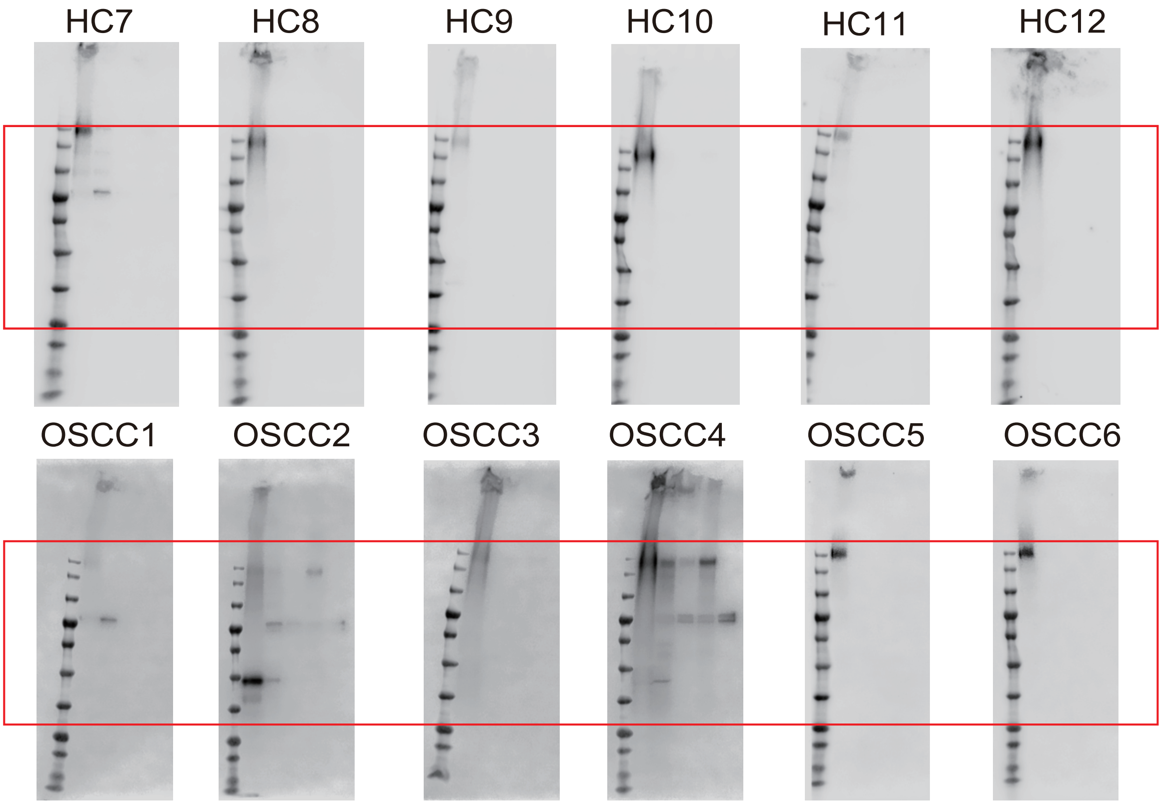

For Fig. 4**a** EGFR

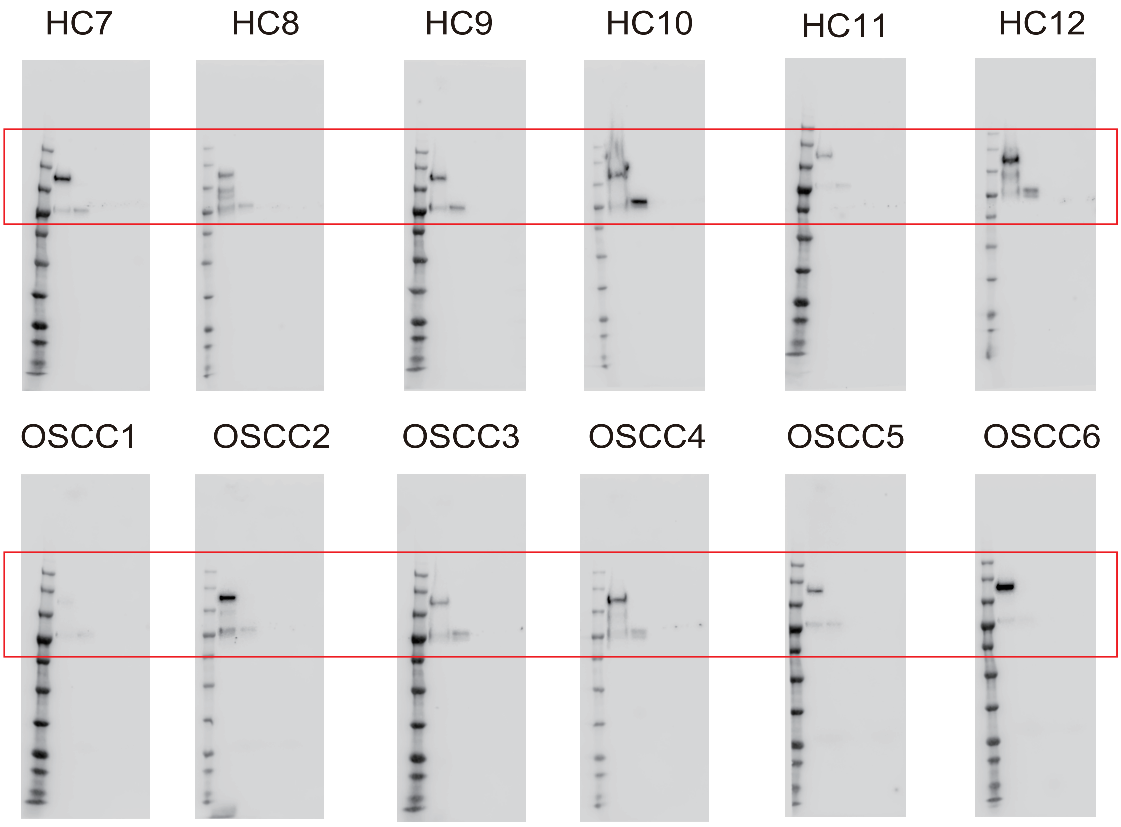

For Fig. 4**a** E-cadherin

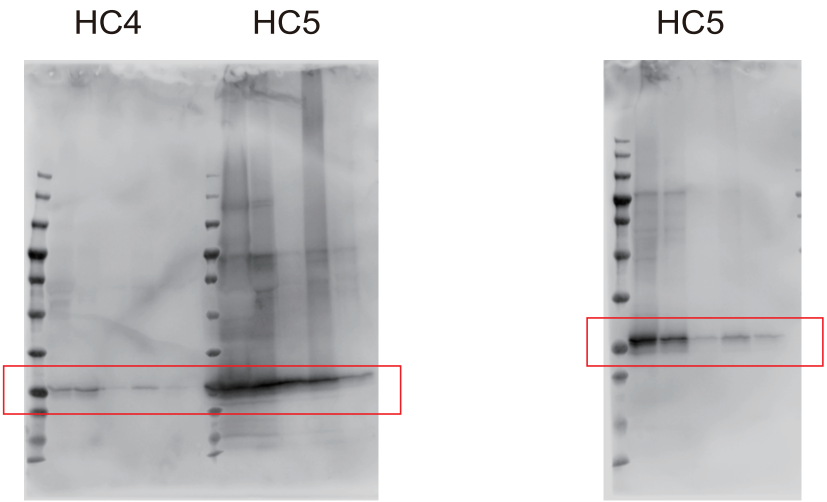

For Fig. S9 Apo-A1

Original data for PCR

HC1

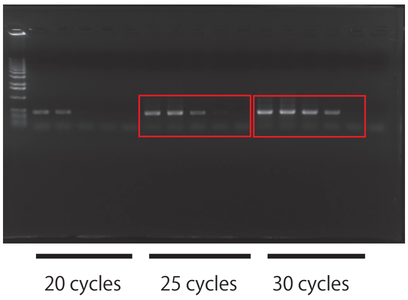

HC2

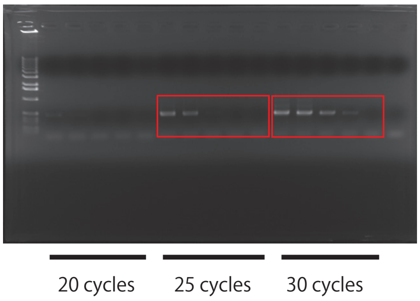

HC3

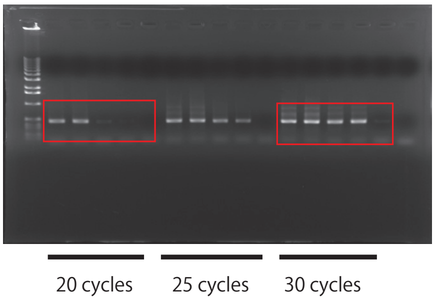

HC4

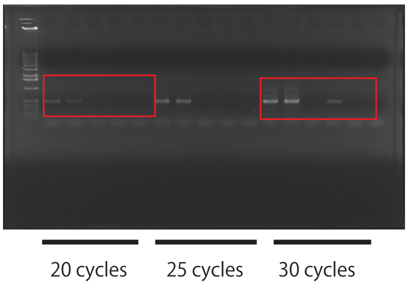

HC5

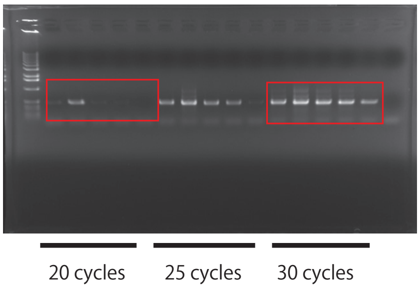

HC6

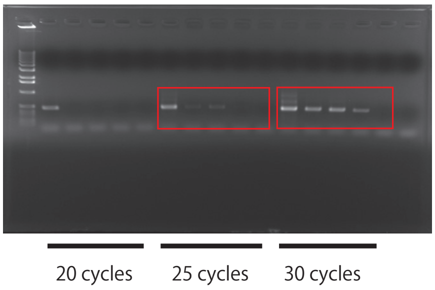

For Fig. S5
